# Development and *in vivo* pharmacokinetic evaluation of a phospholipid complex self-nanoemulsifying drug delivery system (PLC-SNEDDS) for enhanced oral bioavailability of cannabidiol

**DOI:** 10.1080/10717544.2026.2702143

**Published:** 2026-07-22

**Authors:** Thabata Muta, Songhita Mukhopadhyay, Benjamin Noll, Yunmei Song, Sanjay Garg

**Affiliations:** a School of Pharmacy and Biomedical Sciences, Adelaide University, Adelaide, SA, Australia

**Keywords:** Cannabidiol, oral bioavailability, phospholipid complex, pharmacokinetics, self-nanoemulsifying drug delivery system

## Abstract

Cannabidiol (CBD) exhibits poor oral bioavailability (approximately 6%) due to low solubility and excessive first-pass metabolism, limiting its therapeutic potential. This study introduces a novel phospholipid complex self-nanoemulsifying drug delivery system (CBD-PLC-SNEDDS) to enhance CBD delivery. CBD-PLC was integrated into an optimized SNEDDS *via* Design of Experiments (DoE), yielding nanoemulsions with 118.9 ± 0.77 nm particle size, 0.258 PDI, and −21.9 mV zeta potential. Physicochemical characterization (DSC, FTIR) confirmed amorphization and physical encapsulation without chemical alteration. *In vitro* dissolution showed 100% CBD release within 1 h for CBD-PLC-SNEDDS vs. 8 h for CBD-SNEDDS. Stability studies (ICH guidelines) retained 94.73% ± 0.62% CBD at 25 °C/60% RH and 80.21% ± 0.61% at 40 °C/75% RH after 4 months with preservatives. *In vivo* pharmacokinetics in Sprague–Dawley rats (*n* = 9, 20 mg/kg oral; 4 mg/kg IV) demonstrated that CBD-PLC-SNEDDS significantly enhanced systemic exposure, achieving a calculated absolute bioavailability (F) of 92%, compared to 47% for the oleic acid control. The formulation yielded a 5-fold higher *C*_max_ (593 ± 246 vs 118 ± 63 ng/mL) doubled AUC_0-∞_ (88 vs. 45 h·kg·ng/mL/mg), faster *T*_max_ (2 ± 0.3 vs. 7.4 ± 2.3 h), and extended *T*_1/2_ (3.7 ± 0.9 vs. 1.9 ± 0.6 h) versus control. CBD-PLC alone yielded only 39%. IVIVC modelling *via* Wagner–Nelson deconvolution established a strong correlation (R^2^ > 0.7) between *in vitro* dissolution and *in vivo* absorption, validating the system's predictive performance. This synergistic PLC-SNEDDS platform outperforms prior systems, offering a scalable template for lipophilic drugs and paving the way for clinical CBD therapeutics.

## Graphical abstract

**Figure:**
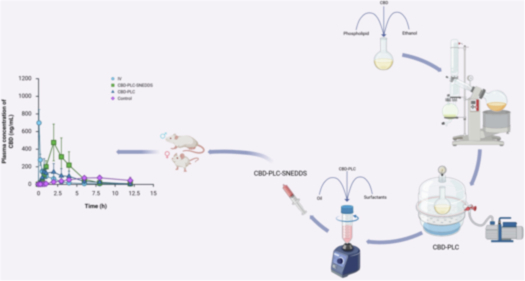

## 1. Introduction

Oral drug delivery remains the most preferred and patient-compliant route of administration (Tong et al., 2019); however, the poor aqueous solubility of many active pharmaceutical ingredients (APIs) continues to hinder their clinical translation. Recent data indicate that approximately 40% of marketed drugs and up to 90% of drug candidates under development exhibit poor water solubility, leading to low oral bioavailability, erratic pharmacokinetics, and suboptimal therapeutic efficacy (Ma et al., 2022; Kumari et al., 2023). Furthermore, over 67% of newly developed chemical entities are poorly water-soluble, and fewer than 8% possess both high solubility and high permeability (Ma et al., 2022).

Cannabidiol (CBD) is a BCS Class II compound with high lipophilicity (log P = 6.3) and extremely low aqueous solubility (~10 µg/mL) (Vlad et al., 2020). Although CBD demonstrates a broad spectrum of pharmacological activities – including analgesic, anti-inflammatory, anxiolytic, antitumor, and neuroprotective effects – it remains underutilized clinically due to its low and inconsistent oral bioavailability, typically estimated at ~6% (Mannila et al., 2007). This limitation is primarily attributed to poor solubility, extensive first-pass metabolism, and potentially physicochemical instability in gastric environments (Millar et al., 2020). While some *in vitro* studies suggest that CBD might be susceptible to degradation under simulated gastric fluid (Merrick et al., 2016), the physiological relevance still remains a topic of debate (Nahler et al., 2017). Furthermore, the extent to which significant CBD degradation occurs in human GI tract remains inconclusive, with recent literature suggesting that CBD conversion to psychoactive cannabinoids like THC doesn't happen to a significant degree *in vivo* (Crippa et al., 2020). In addition, CBD is susceptible to degradation under conditions of heat, light, and oxidation, further complicating its formulation (Millar et al., 2020).

Recent advancements in drug delivery systems have improved CBD’s oral bioavailability. For instance, Nanostructured Lipid Carriers (NLCs) achieved a 4-fold bioavailability increase (27% in rats) *via* optimized lipid matrices (Taha et al., 2025). Similarly, micro-emulsifying capsules (CBDNEXT Supra Capsule) yielded a 5.7-fold higher *C*_max_ in humans (Pisak et al., 2025), while camel milk-derived exosomes enhanced plasma concentrations by 5.75-fold (Aare et al., 2024). Self-Nanoemulsifying Drug Delivery Systems (SNEDDS) and zein nanoparticles have also shown 2.3–2.7 fold bioavailability improvements (Nie et al., 2024; Hermush et al., 2025). These nanotechnology-based approaches highlight the potential to overcome CBD's biopharmaceutical limitations, yet none have achieved near-IV bioavailability. Our novel CBD-PLC-SNEDDS formulation integrates phospholipid complexation with SNEDDS to maximize solubility, stability, and oral absorption, offering a superior platform for CBD delivery.

Phospholipid complexes (PLCs) represent a promising strategy for enhancing the lipophilicity of such poorly water-soluble compounds through non-covalent interactions, including hydrogen bonding and Van der Waals forces, thereby improving their incorporation into lipid-based formulations like SNEDDS (Kuche et al., 2019). This integration facilitates superior drug loading, spontaneous formation of nanoemulsions with droplet sizes typically below 100 nm upon aqueous dispersion, enhanced gastrointestinal stability, membrane permeability *via* modulation of membrane fluidity and transient tight junction opening, ultimately maximizing systemic drug absorption and circumventing excessive hepatic first-pass metabolism (Zhou et al., 2013). Consequently, the synergistic combination of PLCs and SNEDDS can yield multifield improvements in oral bioavailability without altering the inherent permeability profile of BCS Class II drugs (Kazi et al., 2019).

Prior PLC-SNEDDS systems have demonstrated bioavailability enhancements for BCS II and III drugs. For example, silybin-PLC-SNEDDS (1:1 w/w) achieved an 18-fold increase in rats (Tong et al., 2019), paclitaxel-PLC-SNEDDS (1:1 w/w) a 3.42-fold improvement (Ding et al., 2019) and gentiopicroside-PLC-SNEDDS (1:2 molar) a 9.7-fold boost (Tong et al., 2023). Building on our prior CBD-PLC development (Muta et al., 2025), we successfully improved water solubility and permeability through amorphization. In the present study, we hypothesized that integrating this pre-formed complex into a SNEDDS would provide a harmonious effect: the PLC ensures molecular dispersion and improved hydrophilic-lipophilic balance, while the SNEDDS provides the lipidic environment necessary to maximize gastrointestinal absorption and systemic exposure. This two-step rationalization aims to push oral CBD bioavailability toward IV-equivalence, a goal that conventional single-platform delivery systems have yet to achieve. While recent literature reports that optimized SNEDDS can achieve a significant 12.9-fold increase in absolute bioavailability (from 0.3% to 4.4%) for lipophilic compounds, such values remain far below intravenous equivalence, highlighting the need for more advanced integrated platforms (Wang et al., 2020).

To our knowledge, this is the first study to develop and evaluate a CBD-PLC-SNEDDS formulation, including its pharmacokinetic (PK) performance *in vivo,* that has achieved near IV bioavailability through oral delivery. The integration of these two delivery platforms represents a novel approach to overcoming the biopharmaceutical limitations of CBD and could offer a versatile template for other poorly soluble, highly lipophilic drug candidates.

## 2. Materials and methods

### 2.1. Materials

CBD crystals (powder) were provided by Green Dispensary Compounding (Rx518926; Adelaide, Australia). HPLC-grade methanol (1.06018.4000) and acetonitrile (1.00030.2500) were from EMD Millipore® (Billerica, MA, USA). Ultra-pure water was generated using a Sartorius system (Goettingen, Germany). Other reagents: Ethanol (AJA214-10LPL; Thermo Fisher Scientific, Melbourne, Australia); L-α-Phosphatidylcholine (P5394-10G; egg yolk, ≥40%), formic acid (AC10760050), D-chloroform (570699), Tween 60 (P1629-500ML), Cremophor EL (C5135-500G), Kolliphor RH 40 (07076-1KG), Span 80 (S6760-250ML) (Merck Pty Ltd., Sydney, Australia); N-Octanol (OL001-500M), oleic acid (30-1299), Tween 80 (50259531) (ChemSupply, Adelaide, Australia); CBD-D_3_ (C-084-1ML; Novachem Pty Ltd., Melbourne, Australia); Butylated hydroxytoluene (30-1463), Miglyol 812 N (3093), almond oil (0975), acid-resistant CONI-SNAP #00 capsules (3100-09) (Medisca, Sydney, Australia); Polyethylene glycol 400 (GC0231) (Glentham Life Sciences Ltd., Corsham, UK); Captex® 300 (080228-6), Captex® 355 (14026UT14) (ABITEC Corp., Wisconsin, USA); Gelucire® 44/14 (3051PP1), Gelucire® 50/13 (3055), Maisine CC (3431), Transcutol P (3260), Labrasol® ALF (3405) (Gattefossé, Saint-Priest, France). Sorbic acid (30-5174), sesame oil (30-1436-480 ML), and tocopherol (30-1031-25 GM; PCCA, NSW, Australia). Design-Expert® 360 (v23.1.3; Stat-Ease, Minneapolis, MN, USA) was used for DoE; OriginLab Corporation, Northampton, MA, USA, software package were used for data visualization. Biorender (accessed on 6 September 2025) and Biorender Graph's statistical analysis, which uses R (version 4.2.2), was utilized to compute all results of statistical analyses.

### 2.2. Analytical method for CBD quantification

#### 2.2.1. HPLC method for CBD quantification

The analytical method used for CBD quantification was based on the protocol established in our previous study (Muta et al., 2025).

#### 2.2.2. LC-MS method for CBD quantification for pharmacokinetic analysis

The samples were analyzed using a Sciex triple-quadrupole 6500+ LC-MS/MS system (SCIEX, Framingham, MA, USA) in positive electrospray ionization mode. A 5 μL aliquot of each sample was injected using a Shimadzu ultra-high pressure liquid chromatography system (Shimadzu, Kyoto, Japan) onto a Phenomenex Kinetex C18 column (100 × 2.1 mm, 1.7 μm, 100 Å) at a flow rate of 0.2 mL/min, with the column maintained at 40 °C. The mobile phases consisted of ultra-pure water with 0.1% formic acid (A) and methanol with 0.1% formic acid (B). Gradient elution was as follows: 0.1–6.0 min, 2% B; 6.0–7.0 min, 100% B; 8.0–9.0 min, 2% B, for a total run time of 9 min with dwell times of 100 ms and source temperature of 400 °C. Multiple reaction monitoring (MRM) transitions for CBD were 315.1 → 193.1 (quantifier 1) and 315.1 → 259.1 (quantifier 2); for the internal standard CBD-D3, they were 318.1 → 196.1 (quantifier 1) and 318.1 → 262.0 (quantifier 2).

### 2.3. Formulation development and optimization of CBD-PLC-SNEDDS

#### 2.3.1 Initial screening for SNEDDS formulations

A combinatorial library of 56 unique formulations was developed by blending surfactants and oils at a 1:1 (v/v) ratio, as detailed in Table 1. The surfactants and co-surfactants were selected based on their high solubilization capacity for CBD and CBD-PLC. Also, they are widely utilized in lipid-based drug delivery systems due to their low toxicity and excellent ability to reduce interfacial tension, facilitating the spontaneous formation of droplets in the nanometer range. The concentration ranges (levels) for the independent variables were defined based on regulatory safety limits (GRAS/US FDA Inactive Ingredient Database) and reported literature values for successful SNEDDS solidification, ensuring the resulting formulations are both stable and clinically viable (Tong et al., 2019; Ding et al., 2019; Tong et al., 2023; Taha et al., 2025; Pisak et al., 2025; Hermush et al., 2025).

The selection of GRAS (generally recognized as safe) excipients with an optimized hydrophilic-lipophilic balance (HLB) is crucial to prevent drug precipitation during gastrointestinal transit and to enhance absorption, potentially supported by bile salt stimulation (van Hoogevest, 2020). Key parameters assessed during lipid-drug interaction analysis included solubilization capacity, partition coefficient (log P), droplet size, and zeta potential, evaluated using dynamic light scattering and complementary physicochemical methods (Patel et al., 2018; van Hoogevest, 2020). Each formulation, prepared at a total volume of 3 mL, was aliquoted into vials to evaluate miscibility and physicochemical compatibility. Achieving isotropic mixtures of oils, surfactants, and co-surfactants is essential for effective initial screening, ensuring uniform dispersion and stability (Patel et al., 2018).

Homogenization was performed *via* continuous vortex agitation for 3 h at maximum speed using a multitube vortex mixer (Model MTV1, Ratek, Melbourne, Australia). Formulations with inadequate homogeneity underwent an additional 10-s manual vortexing to ensure consistency.

To facilitate emulsification of formulations containing solid or semi-solid surfactants (e.g. Gelucire® 44/14 and Gelucire® 50/13), vials were incubated at 60 °C for 24 h in a precision thermoregulated oven (Axyos, Queensland, Australia) to promote liquefaction and enhance molecular dispersion. Post-incubation, formulations were re-vortexed for 60 s to ensure uniform phase integration (Nasr et al., 2016).

#### 2.3.2. Phase separation analysis

To evaluate thermodynamic stability, 1 mL aliquots of each formulation were transferred to 1.5 mL polypropylene tubes and subjected to high-speed centrifugation at 16,100 × *g* for 15 min at 23 °C using a Microfuge 16 (Beckman Coulter, Brea, CA, USA). Post-centrifugation, samples were inspected visually for evidence of phase separation, creaming, or sedimentation, indicative of colloidal instability (Singh and Pai, 2015).

#### 2.3.3. Accelerated stability assessment

All 56 formulations underwent accelerated stability testing at 60 °C within a thermoregulated oven (Kim et al., 2017). Stability was monitored at 1- and 2-week intervals through macroscopic evaluation of chromatic shifts, turbidity, or phase disjunction. Formulations demonstrating robust stability after 14 days were advanced by incorporating four distinct co-surfactants, generating an additional 16 formulations. These derivative systems were subjected to identical centrifugation conditions (16,100 × *g*, 15 min, 23 °C) to ascertain phase integrity and colloidal stability.

#### 2.3.4. Optimization strategy (Nasr et al., 2016)

Formulations exhibiting sustained stability underwent extended evaluation under accelerated conditions (60 °C) for an additional 14 days to identify the most robust oil-surfactant-co-surfactant matrix. The lead formulation was optimized using a Design of Experiments (DoE) approach to systematically explore surfactant-to-oil ratios, targeting SNEDDS optimized for scalable production and enhanced oral bioavailability of CBD.

**Table 1.** Surfactants, co-surfactants, and oils evaluated in the initial screening for CBD-PLC-SNEDDS formulations.

Excipient name	Inactive ingredient database (UNII)	Function in formulation	Reference
Cremophor EL	6D4M1DAL6O	Surfactant, co-surfactant	U.S. Food and Drug Administration (FDA), (2025)
Labrasol® ALF	00BT03FSO2		
Tween 60	CAL22UVI4M	Surfactant	
Span 80	06XEA2VD56		
Gelucire® 44/14	H5ZC52369M		
Gelucire® 50/13	G6EP177239		
Tween 80	6OZP39ZG8H		
Kolliphor RH 40	7YC686GQ8F		
Transcutol P	A1A1I8X02B	Co-surfactant	
PEG 400	B697894SGQ		
Oleic acid	2UMI9U37CP	Oil	
Captex® 355*	C9H2L21V7U		
Captex® 300*	C9H2L21V7U		
Sesame oil	QX10HYY4QV		
Almond oil	66YXD4DKO9		
Miglyol 812 N	C9H2L21V7U		
Maisine® CC	4763AXI84L		

#### 2.3.5. Preparation of CBD-PLC-SNEDDS

Briefly, CBD-PLC was optimized and prepared using the solvent evaporation method. Predetermined amounts of CBD and L-α-Phosphatidylcholine from dried egg yolk were dissolved in 30 mL of ethanol and stirred at 40 °C for 30 min. The solvent was removed *via* rotary evaporation under reduced pressure, yielding a solid product that was further dried under vacuum at room temperature overnight to ensure the removal of residual solvent. The resulting CBD-PLC was then stored at 4 °C in a sealed container prior to its incorporation into the SNEDDS (Muta et al., 2025): CBD-PLC was accurately weighed into a flask, followed by the addition of SNEDDS. The mixture was vortexed at maximum speed for 1 min, sonicated for 20 min, and vortexed again for 1 min. This cycle of vortexing (1 min) and sonication (20 min) was repeated three times to achieve complete dissolution of CBD-PLC in SNEDDS. In some instances, manual mixing with a spatula was employed to facilitate homogenization and minimize the duration of sonication and vortexing (Eid and Elmarzugi, 2019).

#### 2.3.6. Experimental optimization using design of experiments

A statistically driven DoE approach was employed to optimize the formulation of CBD-PLC-SNEDDS using Design-Expert® software (Stat-Ease, Minneapolis, MN, USA). An I-optimal (D-optimal custom) mixture design was implemented to systematically explore excipient combinations while ensuring each formulation comprised at least one surfactant, one co-surfactant, an oil phase, and the CBD-PLC. Eight components were evaluated: Kolliphor® RH40 (A), Labrasol® ALF (B), Tween® 60 (C), Transcutol® HP (D), polyethylene glycol (PEG) 400 (E), Cremophor® EL (F), oleic acid (G), and CBD-PLC (H). Component proportions were constrained based on preliminary solubility and miscibility assessments, with the total composition summing to 100% w/w.

The experimental matrix consisted of 41 runs, incorporating model points and lack-of-fit points. Critical quality attributes selected as response variables included particle size (R1), polydispersity index (PDI) (R2), and zeta potential (R3), which are pivotal for nanoemulsion stability and bioavailability. Response data were analyzed using analysis of variance (ANOVA) to validate predictive models. Multi-response numerical optimization was performed to identify the optimal formulation based on desirability criteria, targeting minimized particle size (<200 nm), PDI < 0.3, and zeta potential magnitudes exceeding ±30 mV to enhance colloidal stability and oral absorption of CBD (Mohd Izham et al., 2019).

### 2.4. Physicochemical characterization

#### 2.4.1. Particle size, polydispersity index, zeta potential, and transmittance

Formulations were emulsified by diluting the CBD-PLC-SNEDDS preconcentrate with reverse osmosis (RO) water at a 1:10 (v/v) ratio, followed by an additional 1:10 dilution after filtration through a 0.2 µm cellulose acetate syringe filter (Sartorius). The resultant nanoemulsions were characterized for hydrodynamic particle size, PDI, zeta potential, and transmittance using dynamic light scattering (Zetasizer Nano ZS, Malvern, UK) and UV-Vis spectroscopy (Evolution 201, ThermoFisher, Shanghai, China) to evaluate colloidal stability and optical clarity (Baloch et al., 2019).

Spectroscopic properties were assessed by dissolving 5 mg of each formulation in RO water, followed by analysis using a UV-Vis spectrophotometer over a wavelength range of 200–800 nm to determine absorbance profiles.

#### 2.4.2. Encapsulation efficiency

Approximately 10 mg of CBD-PLC-SNEDDS was transferred into a 1.5 mL polypropylene tube and extracted with 1 mL of methanol. The mixture was vortexed at low speed for 1 h using a multi-tube vortex mixer (Ratek, Australia). Following extraction, samples were centrifuged at 16,100 rcf for 20 min to obtain a clear supernatant, which was subsequently filtered through a 0.45 µm PVDF syringe filter. Quantitative analysis was performed, and results were calculated using the equations outlined below (Muta et al., 2025).

$$\mathrm{EE}\;\left(\%\right)=\frac{\mathrm{Total}\;\mathrm{CBD}\;\mathrm{added}-\mathrm{Unentrapped}\;\mathrm{CBD}}{\mathrm{Total}\;\mathrm{CBD}\;\mathrm{added}}\times 100.$$

#### 2.4.3. Differential scanning calorimetry (DSC)

DSC analysis was conducted using a DSC250 instrument (TA Instruments, DE, USA) to determine the onset temperature, melting point, width of melting events (WME), enthalpy, and crystallinity index (CI) of the physical mixture (PM) and CBD-PLC-SNEDDS. Approximately 3 mg of each sample was sealed in an aluminum pan and heated from 25 to 200 °C at a rate of 10 °C/min (Muta et al., 2025). Nitrogen was employed as the purge gas at a flow rate of 50 mL/min. The WME and CI were calculated according to the following equations (Muta et al., 2025):

WME(°C)=meltingpoint−onsettemperature,

CI(%)=(ΔHBM/ΔHSL)×100.

The PM was prepared by accurately weighing and manually blending pure CBD with the formulation excipients (L-*α*-Phosphatidylcholine, oleic acid, Kolliphor RH40, Tween 60, and PEG 400) in the same ratios as the optimized CBD-PLC-SNEDDS. This mixture was prepared without the use of solvents, heat, or high-energy mixing to serve as a crystalline reference for DSC and FTIR analyses.

#### 2.4.4. Fourier-transform infrared spectroscopy (FTIR) and principal component analysis (PCA)

FT-IR spectroscopy was utilized to examine potential molecular interactions between CBD and phospholipid components in the SNEDDS formulation, compared to the PM and pure CBD. Spectra were recorded using a Tensor 27 infrared spectrophotometer equipped with a Specac Golden Gate attenuated total reflectance (ATR) module (Bruker, Ettlingen, Germany), scanning from 4000 to 400 cm^−1^ at 4 cm^−1^ resolution and 64 scans per spectrum. Samples (~5 mg) were placed directly on the ATR crystal for analysis. Data processing involved baseline correction and normalization using appropriate software (OriginPro).

Key regions analyzed included O─H/N─H stretching (4000–3000 cm^−1^), C─H stretching (3000–2800 cm^−1^), C═O stretching (1800–1650 cm^−1^), aromatic C═C (1650–1500 cm^−1^), and the fingerprint region (1500–400 cm^−1^) (Muta et al., 2025).

PCA was applied to the FT-IR spectra to explore variances and differentiate between samples using OriginPro. Data were mean-centered, and principal components were extracted based on eigenvalues ≥ 1, focusing on the first two components to capture the majority of variance. The score plot was generated to visualize sample clustering and separation (Muta et al., 2025).

### 2.5. *In* vitro dissolution testing

Dissolution studies were performed using the paddle method (USP Apparatus II) in accordance with the United States Pharmacopoeia (USP 35). Each assay was conducted in 90 mL of RO water (pH 5.6) contained in a small vessel (Muta et al., 2025), which was immersed in a water bath maintained at 37 °C ± 0.5 °C. The paddle rotation speed was set at 100 rpm. Approximately 900 ± 50 mg of each formulation, containing equivalent amounts of CBD, was filled into acid-resistant size #00 capsules and carefully placed onto the surface of the dissolution medium. To ensure consistent hydrodynamics and mitigate potential vortex effects at the 100 rpm rotation speed, each capsule was fitted with a USP-compliant sinker to maintain a fixed position at the bottom of the vessel. This setup facilitates the rapid, spontaneous emulsification required for the CBD-PLC-SNEDDS while maintaining uniform exposure to the dissolution medium. 3 mL were withdrawn at predetermined intervals (0, 0.25, 0.5, 0.75, 1.0, 2.0, 3.0, 4.0, 6.0, 8.0, 12.0, and 24.0 h), with an equal volume of fresh medium replaced after each sampling to maintain sink conditions. Samples were filtered through a 0.45 µm membrane filter, and 20 µL of the filtrate was injected into the HPLC system for quantitative analysis (Muta et al., 2025).

### 2.6. Stability studies

Stability studies were conducted in accordance with ICH Q1A(R2) guidelines (Nasr et al., 2016). CBD-PLC-SNEDDS formulations (10 mg each) were transferred into glass vials to ensure light protection and stored for 4 months under two conditions: intermediate (25 °C, 60% relative humidity, light-protected) and accelerated (40 °C, 75% relative humidity, light-protected). At predetermined intervals (0, 1, 2, 3, and 4 months), 10 mg aliquots were withdrawn, diluted with 1 mL of HPLC-grade methanol, vortex-mixed for 30 s at 2000 rpm, filtered through a 0.45 µm PVDF membrane, and analyzed by HPLC using the validated method described in Section 2.2 to quantify CBD content.

### 2.7. Pharmacokinetic evaluation

#### 2.7.1. *In vivo* studies

All surgical and experimental procedures received approval from the Animal Experimentation Ethics Committee of the University of South Australia, Adelaide, Australia. The crossover study design (*n* = 9 total: 5 males, 4 females - Supplementary Table S1) used in this study serves to reduce the number of rats required; subjects act as their own control partially, treatment replication is balanced across subjects (*n* = 3 per treatment), and are sequenced to minimize order effects, includes contingency and the design is duplicated for each sex. During experimental periods, Sprague–Dawley rats (Ozgene ARC Pty Ltd, Innaloo, WA, Australia) 250 ± 50 g) were housed individually in Culex^®^ cages under standard conditions in a temperature-controlled facility with a 12-h light/dark cycle and free access to food, water and limited environmental enrichment. Inclusion criteria mandated that all rats were age-matched (6–7 weeks) and habituated to the facility for at least 5 days post-arrival. On the other hand, exclusion criteria were defined by unresolved surgical complications; rats were removed from the cohort if they exhibited loss of cannula patency or excessive blood loss that could compromise physiological integrity or data consistency.

To minimize bias, a single-blind approach was implemented during the dosing phase. One researcher was responsible for the preparation of all formulations (CBD-PLC-SNEDDS, CBD-PLC, and control). A second researcher, who was not involved in the formulation preparation and was unaware of the specific composition of each group, performed the animal dosing. The formulations were provided to the dosing researcher labelled only as ‘Formulation A,’ ‘Formulation B,’ ‘Control,’ and ‘IV’ to ensure the administrator remained blinded to the treatment identity during the conduct of the experiment.

Whole blood samples were collected from rats using a Culex® Automated Blood Collection System (BASi, West Lafayette, IN, USA). Each animal was surgically implanted with an indwelling catheter in either the carotid artery or jugular vein under anesthesia and were allowed to recover overnight prior to dosing. Blood (~50 µL) was collected into pre-heparinized tubes at eleven pre-determined time points (0.25, 0.5, 0.75, 1, 2, 3, 4, 6, 8, 12, and 24 h after an oral dose; 0.08, 0.25, 0.5, 1, 2, 3, 4, 6, 8, 12, and 24 h after an intravenous dose), centrifuged (16,100 rcf for 5 min at 4 °C) to separate plasma and stored at −20 °C until analysis. Subsequent doses were administered 24 h after collection of the final (24 h) sample from the preceding dose.

To evaluate the bioavailability of CBD, pre-concentrates of CBD-PLC were vortex-mixed in water, forming oil-in-water (O/W) nano-dispersions with a CBD concentration of 5 mg/mL. These formulations and control (CBD in oleic acid at a concentration of 5 mg/mL) were administered *via* oral gavage to rats (*n* = 5–6 per group) at a CBD dose of 20 mg/kg. For intravenous (IV) administration, CBD was delivered in a vehicle of propylene glycol:ethanol:water (8:1:1, v/v/v) (Cherniakov et al., 2017; Kok et al., 2022) at 4 mg/mL and a dose of 4 mg/kg, into the tail vein.

Animal welfare was monitored daily using a weighted clinical scoring system (0-4) assessing behavioral and physiological markers, with humane endpoints defined by a cumulative score of ≥4 respiratory distress, or acute weight loss exceeding 15%. Once the study endpoint has been reached, the rats were humanely killed by cervical dislocation under full anesthesia by carbon dioxide inhalation or by the administration of either Pentabarbitone or Lethabarb as an alternative to cervical dislocation. Since the rats have a cannula implanted into either the jugular vein or carotid artery, the humane killing agents can be administered directly through that route:

1. Administer 200 mg/kg of pentobarbitone 325 mg/mL (Lethabarb; neat) via the cannula (either conscious or under general anesthesia the cannula cannot be accessed, or patency has been compromised:
2. Administer 200 mg/kg of pentobarbitone 325 mg/mL (Lethabarb; neat) intra-cardiac under general anesthesia. Or;
3. Administer 200 mg/kg of Lethabarb diluted to 60 mg/mL (1:4 dilution of Lethabarb/Normal Saline) by intra-peritoneal injection. If the animal is still conscious after 5 min repeat the injection.

#### 2.7.2 Plasma sample preparation and LC-MS/MS analysis

Plasma aliquots (20 µL) were transferred to 1.5 mL polypropylene tubes and spiked with 2.5 µL of internal standard (CBD-D_3_, 100 µg/mL in methanol). Samples were vortex-mixed for 30 s and equilibrated at room temperature for 30 min. CBD extraction was performed by adding 77.5 µL of ice-cold acetonitrile, followed by vortex-mixing for 30 s and incubation at −20 °C for 30 min to ensure complete protein precipitation. Samples were then centrifuged at 16,100 rcf for 20 min, and 50 µL of the supernatant was transferred to vials for LC-MS/MS analysis.

#### 2.7.3. Pharmacokinetic analysis

PK analysis was conducted using Phoenix 64® WinNonlin® Version 8.3.5.340 (Certara™, NJ, USA). PK parameters were determined using non-compartmental methods (Model Type: Plasma 200–202) and a linear-trapezoidal linear interpolation approach. Plasma parameters, namely Area under plasma concentration time curve from time of dosing extrapolated to infinity (AUC_0-∞_ dose normalized), maximum plasma concentration (*C*_max_), time at which maximum concentration was achieved (*T*_max_), and elimination half-life *T*_1/2_ were considered for final analysis.

To facilitate an accurate comparison between the intravenous (4 mg/kg) and oral (20 mg/kg) administration routes, all pharmacokinetic parameters involving systemic exposure were dose-normalized. Absolute bioavailability (*F*) was calculated by dividing the dose-normalized AUC_0-∞_ of the oral formulations by the dose-normalized AUC_0-∞_ of the IV bolus. This normalization is a standard methodology that accounts for dose variations, ensuring that the calculated *F* value for the oral formulations accurately represents the fraction of the administered dose reaching systemic circulation (Certara, 2026).

#### 2.7.4. Statistical analysis for PK

Data are presented as mean ± standard deviation (SD) unless otherwise specified. Statistical significance between experimental groups was assessed using a two-tailed *t*-test or one-way ANOVA followed by Kruskal–Wallis's test, with a *p*-value < 0.05 considered significant.

### 2.8. *In-vitro in-vivo* correlation (IVIVC)

IVIVC modelling was performed using the deconvolution technique based on the Wagner–Nelson method.

Gonzalez and Smith (2015) implemented in Origin(Pro), Version (10.2.0.188) 2025; OriginLab Corporation, Northampton, MA, USA software package. *In vivo* plasma concentration data from animal studies pre-processed in Phoenix 64® WinNonlin® Version 8.3.5.340 (Certara™) software, for area under the curve (AUC_last_) for each time point and the total AUC to infinity (AUC_0-∞_) were calculated using the trapezoidal rule.

The relative fraction absorbed was calculated from this *in vivo* data using the Wagner–Nelson equation as depicted below:

$$F\left(t\right)=\frac{C\left(t\right)+\mathrm{ke}\times \mathrm{AUClast}}{\mathrm{ke}\times \mathrm{AUC}0-\infty }.$$

*C*(*t*): Plasma concentration of CBD from CBD-PLC-SNEDDS at time *t.*

*K_e_*: Elimination rate constant typically determined from the terminal phase of the plasma concentration time curve.

AUC_last_: Area under the plasma-concentration curve from time 0 to time *t.*

AUC_0-∞_: Area under the plasma-concentration curve from time 0 to infinity.

The above fraction was converted to a percentage to give the percentage of the relative fraction absorbed (Davanço et al., 2020). The data of Time point vs Percentage relative fraction absorbed was imported to Origin, and a Wagner–Nelson plot was created to demonstrate the conversion of a typical plasma concentration profile into an absorption profile labelled as ‘Wagner–Nelson plot’ in Figures 12 and 13.

To generate IVIVC, post-development of the Wagner Nelson plot, the data were further analyzed to calculate the absorption rate constant by developing a semilogarithmic plot. This was achieved by calculating the percentage relative fraction unabsorbed. This parameter was set on the Y-axis (set as Log_10_ scale) and Time on the X-axis, followed by Linear fitting to obtain the slope for further calculation of the absorption rate constant (Ka). Mathematical integration of the absorption profile was performed in OriginLab, followed by non-linear curve fitting analysis to evaluate model suitability. The Logistic model (under Growth/sigmoid category) was selected to demonstrate the model's suitability.

To establish a comparison between *in vitro* dissolution and deconvoluted *in vivo* data, the Exp1p1 model (a one-parameter exponential function) was selected, as *in vitro* dissolution data are often predicted to follow an exponential release trend for controlled-release formulations. Model fit was assessed using the Adjusted *R* ^2^ value to establish the foundation for a reliable IVIVC, which correlates the entire *in vitro* dissolution profile with the *in vivo* absorption profile.

## 3. Results

### 3.1. Formulation development and optimization of CBD-PLC-SNEDDS

#### 3.1.1. Initial screening for SNEDDS development

The initial miscibility screening evaluated 56 surfactant–oil mixtures, with 28 (50%) found to be immiscible in a 1:1 ratio. Stability assessments conducted after 1 week revealed that nine mixtures (16.1%) exhibited no visible phase separation or color change. After 2 weeks at 60 °C, only four mixtures (7.1%) remained stable (shown in Supplementary Figure 1).

**Figure 1.**
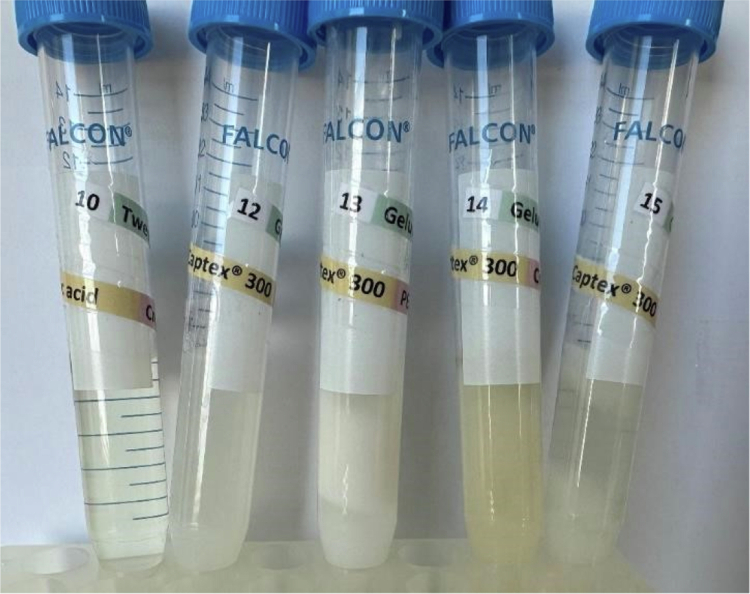
Visual evidence of phase separation in failed CBD-PLC-SNEDDS formulations (10, 12, 13, 14, 15) after centrifugation (3000 rcf, 15 min, 23 °C), compared to stable formulations (Table 2)

These four stable mixtures were combined with four co-surfactants (Kolliphor RH40, Cremophor EL, Labrasol® ALF, Tween 60, Transcutol P, or PEG 400), yielding 15 mixtures due to the dual functionality of Labrasol® ALF as both a surfactant and co-surfactant. Post-centrifugation stability testing (Figure 1) identified phase separation in 5 mixtures. The remaining 10 (Table 2) underwent extended stability testing to select the most robust combination for further optimization using a DoE approach. The selected components included Kolliphor RH40, Labrasol® ALF, Tween 60, Transcutol P, PEG 400, and oleic acid. Results for particle size, PDI, zeta potential, and transmittance of the 41 DoE-generated formulations are presented in Table 3.

**Table 2.** Compositions of formulations passing initial miscibility and centrifugation stability tests for DoE optimization.

No	Surfactant	Oil	Cosurfactant
1	Kolliphor RH40	Oleic acid	Transcutol P
2	Kolliphor RH40		PEG 400
3	Kolliphor RH40		Cremophor EL
4	Kolliphor RH40		Labrasol® ALF
5	Labrasol® ALF		Transcutol P
6	Labrasol® ALF		PEG 400
7	Labrasol® ALF		Cremophor EL
8	Tween 60		Transcutol P
9	Tween 60		PEG 400
11	Tween 60		Labrasol® ALF

**Table 3.** Physicochemical properties (particle size, PDI, zeta potential, and transmittance) of 41 DoE-generated CBD-PLC-SNEDDS formulations.

Sample	Kolliphor RH40 (% w/w)	Labrasol® ALF (% w/w)	Tween 60 (% w/w)	Transcutol P (% w/w)	PEG 400 (% w/w)	Cremophor EL (% w/w)	Oil (% w/w)	CBD-PLC (% w/w)	Particle size (nm)	PDI	Zeta Potential (mV)	Transmittance (%)
1	4.346	4.630	36.846	5.813	12.667	0.000	30.698	5.000	426.467	0.288	−17.73	8.23
2	35.567	23.489	0.000	3.326	0.000	15.308	11.602	10.709	193.967	0.314	−16.53	80.08
3	0.000	30.532	11.306	0.000	0.000	17.357	25.804	15.000	702.267	0.336	−18.6	8.18
4	41.882	0.000	0.000	0.000	10.286	14.239	24.788	8.805	145.767	0.418	−16.77	56.37
5	8.652	38.657	0.000	24.708	0.000	0.000	18.683	9.301	298.1	0.286	−24.93	1.98
6	0.000	55.020	0.000	0.000	5.525	24.455	10.000	5.000	174.767	0.395	−12.4	77.42
7	0.000	50.873	0.000	0.000	10.000	0.000	30.136	8.990	470.233	0.502	−27.13	0.44
8	33.824	24.488	1.688	0.000	21.253	2.328	11.420	5.000	206.433	0.363	−11.97	71.42
9	27.412	0.196	16.439	16.098	0.000	13.902	20.953	5.000	231.767	0.456	−14.7	54.76
10	30.352	2.853	0.000	15.568	14.432	0.000	27.929	8.866	124.667	0.442	−26.97	92.01
11	14.169	23.055	20.652	18.096	0.000	0.000	19.029	5.000	287.967	0.283	−18.27	59.57
12	0.000	0.000	55.000	0.000	30.000	0.000	10.000	5.000	230.9	0.468	−9.73	82.18
13	22.775	0.000	7.225	2.312	0.000	27.688	25.000	15.000	212.833	0.502	−13.37	42.78
14	2.175	0.000	32.121	11.732	0.000	2.106	40.000	11.866	486.067	0.264	−21.5	9.61
15	0.000	0.000	30.000	30.000	0.000	0.000	35.000	5.000	980.9	0.419	−19.77	0.42
16	21.570	12.169	10.808	11.220	14.076	4.704	10.453	15.000	420.667	0.517	−15.73	71.42
17	0.000	30.000	0.000	7.532	11.500	10.968	25.000	15.000	814.7	0.219	−21.03	0.39
18	0.000	41.667	13.333	16.644	13.356	0.000	10.000	5.000	162.833	0.31	−12.6	75.25
19	32.702	0.000	27.298	0.000	8.608	7.725	12.577	11.090	251.333	0.539	−13.8	80.96
20	3.357	51.643	0.000	0.000	24.098	5.902	10.000	5.000	168.8	0.265	−12.23	77.99
21	7.123	25.344	0.000	11.619	0.597	10.317	40.000	5.000	341.433	0.498	−26.40	43.76
22	18.724	16.995	0.000	30.000	0.000	0.000	29.281	5.000	496.433	0.397	−27.93	65.54
23	16.745	5.468	37.788	21.902	0.000	0.000	10.000	8.098	147.1	0.243	−12.8	80.45
24	0.000	60.000	0.000	14.826	0.000	0.174	10.000	15.000	323.367	0.544	−27.30	96.60
25	60.000	0.000	0.000	13.887	0.000	0.000	21.113	5.000	106.667	0.167	−17.43	76.83
26	45.000	0.000	0.000	0.000	30.000	0.000	10.000	15.000	114.733	0.435	−7.72	85.94
27	0.000	0.000	60.000	3.169	0.000	18.792	13.039	5.000	187.767	0.603	−12.83	86.89
28	0.000	0.000	60.000	0.000	10.000	0.000	15.000	15.000	149.067	0.373	−14.43	79.94
29	22.950	0.000	7.050	0.000	15.000	0.000	40.000	15.000	113.7	0.288	−24.90	97.78
30	0.000	30.000	0.000	0.000	30.000	0.000	35.000	5.000	103.7	0.27	−39.63	86.72
31	0.000	20.591	27.553	12.570	0.000	17.430	10.000	11.856	286	0.574	−21.10	53.25
32	46.335	3.183	5.482	0.000	0.000	30.000	10.000	5.000	97.5	0.602	−16.70	96.36
33	32.516	0.000	12.484	0.000	0.000	10.000	40.000	5.000	240.2	0.468	−21.83	84.12
34	0.201	16.957	16.122	0.000	11.412	0.319	39.989	15.000	582.1	0.18	−17.60	0.51
35	45.000	0.000	0.000	26.600	0.000	3.400	10.000	15.000	179.6	0.461	−21.90	85.51
36	4.250	0.000	34.019	16.364	8.916	0.000	21.450	15.000	400.2	0.274	−16.70	45.64
37	33.933	14.522	1.285	10.630	0.000	0.000	24.630	15.000	234.2	0.487	−28.00	98.44
38	10.245	7.510	27.245	0.000	14.540	15.460	10.000	15.000	175.5	0.336	−19.77	87.54
39	0.000	33.307	26.693	2.847	0.000	9.829	22.324	5.000	646.7	0.485	−15.17	36.55
40	0.020	0.312	29.668	0.000	7.512	22.488	32.867	7.133	319.9	0.304	−22.70	43.80
41	0.000	35.641	16.775	0.039	22.738	0.000	12.760	12.047	276.7	0.366	−12.60	63.07

#### 3.1.2. Optimization strategy

**Figure 2.**
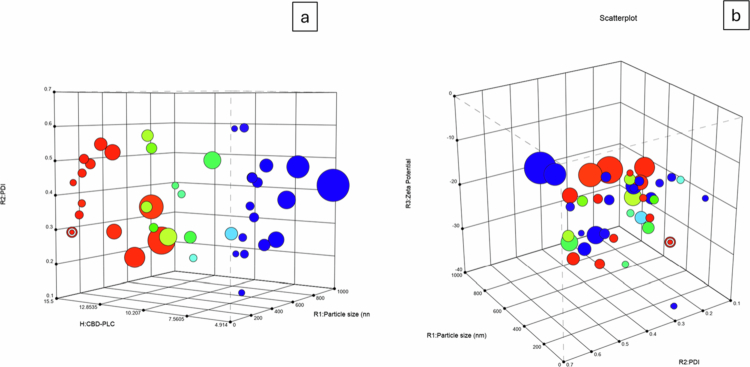
Design of experiments (DoE) optimization plots for CBD-PLC-SNEDDS: (a) 3D surface plot showing the effect of CBD-PLC content (H) on particle size (R1) and PDI (R2). (b) 3D scatterplot illustrating the correlation between particle size (nm), PDI, and zeta potential (mV) across the 41 experimental runs. The red circle highlights the optimized sample 29, which achieved a superior balance of high drug loading (15.45%) and optimal colloidal stability (113.7 nm particle size; −24.9 mV zeta potential).

Among the tested oils, oleic acid was selected for its superior miscibility and stability in initial screening (Section 2.3.1). At 40% w/w, oleic acid enabled formulations targeting particle sizes <200 nm, PDI < 0.3, and CBD-PLC content of 15%, critical for maximizing CBD encapsulation efficiency (>95%) despite the low CBD-to-phospholipid ratio (0.68:20) in the complex (Muta et al., 2025). As shown in Figure 2, Sample 29 achieved the highest CBD-PLC content (15.45%) with a particle size of 113.7 nm and PDI of 0.288, meeting predefined criteria for optimal nanoemulsion. Additionally, Sample 29 exhibited a zeta potential between −30 and −40 mV, indicating enhanced colloidal stability (Figure 2).

**Figure 3.**
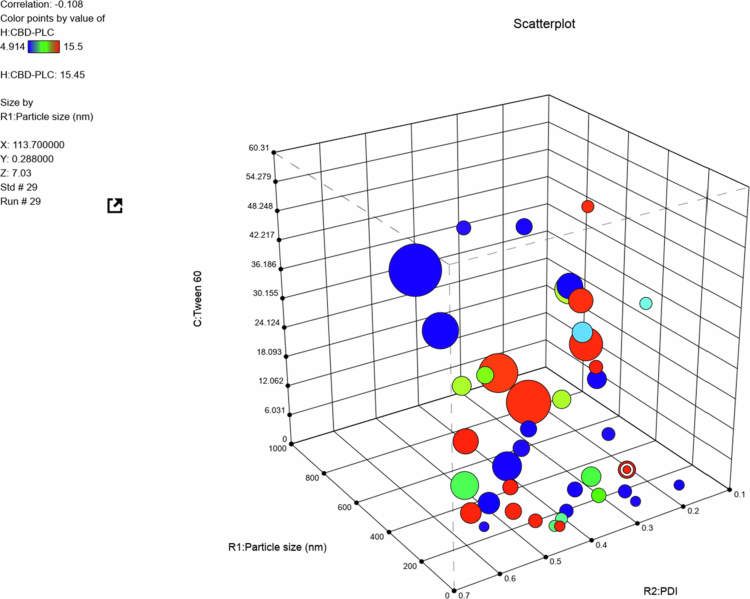
DoE contour plot of sample 29 (red) in CBD-PLC-SNEDDS: high CBD-PLC content, minimal Tween 60, particle size < 0.3.

An additional objective was to minimize Tween 60 concentration to avoid exceeding the maximum daily exposure limits set by US FDA inactive ingredient guidelines (U.S. Food and Drug Administration (FDA), 2025), as high surfactant levels could necessitate larger CBD-PLC-SNEDDS doses to achieve therapeutic CBD levels. Sample 29 optimally balanced high CBD-PLC content with acceptable Tween 60 levels (Figure 3). The DoE software provided a single optimal solution, detailed in Table 4, which closely aligned with Sample 29's composition.

**Table 4.** Excipient proportions and physicochemical parameters of DoE-optimized solution 1 vs. sample 29 for CBD-PLC-SNEDDS.

Sample	Kolliphor RH40 (% w/w)	Labrasol®ALF (% w/w)	Tween 60 (% w/w)	Transcutol HP (% w/w)	PEG 400 (% w/w)	Cremophor EL (% w/w)	Oleic acid (% w/w)	CBD-PLC (% w/w)	Particle size (nm)	PDI	Zeta potential (mV)
29	23.22	0.00	7.03	0.00	16.31	0.00	40.72	15.45	113.7	0.288	−24.9
Solution	11.00	0.00	2.50	0.00	26.00	0.00	39.00	15.50	118.9	0.258	−21.9

To further optimize CBD-PLC loading, the formulation was adjusted to incorporate 21.5% CBD-PLC, exceeding the initial DoE range (0%–15%) based on prior studies (Zhang et al., 2015; Shen et al., 2016; Tong et al., 2019), which demonstrated minor impacts on particle size and PDI across Sample 29 and comparators. This adjustment was supported by literature indicating that elevated phospholipid content enhances colloidal stability *via* stronger intermolecular interactions but may impair dispersion and fluidity at drug-to-phospholipid ratios ≥ 1:3 due to increased viscosity and reduced emulsification spontaneity (Zhou et al., 2013). For instance, Tong et al. observed that a 1:10 weight ratio of gentiopicroside-phospholipid complex (GTP-PC) to blank SNEDDS yielded stable microemulsions with minimal particle sizes in phosphate-buffered saline and hydrochloric acid, whereas higher drug loadings elevated particle size and diminished emulsification efficiency (Tong et al., 2023). At our CBD: phospholipid ratio of ~1:30 (within CBD-PLC) and overall loading of 21.5% (equivalent to ~1:5 CBD-PLC to blank SNEDDS), these risks were mitigated, as evidenced by sustained nanoemulsion characteristics (Table 5).

In contrast, most drug-PLC-SNEDDS studies employ drug-to-phospholipid ratios of 1:1 to 1:5 (Wu et al., 2014; Shen et al., 2016; Ding et al., 2019; Ryšánek et al., 2025) prioritizing higher complex densities that may compromise scalability. To validate our optimized ratios' superior balance of loading and performance, a comparative formulation analysis was conducted (Section 3.1.3, Table 5).

#### 3.1.3. Comparative formulation analysis

To validate the DoE-optimized formulation, the solution provided in Table 4 was compared with three alternative formulations to confirm its superiority over conventional approaches (Wu et al., 2014; Shen et al., 2016; Ding et al., 2019; Ryšánek et al., 2025):

1. CBD-PLC in SNEDDS: CBD-PLC (1:30, w:w) incorporated at the SNEDDS DoE-optimized;
2. CBD-PLC in SNEDDS: CBD-PLC (1:1, w:w) ratio, commonly reported in the literature in drug: PLC ratio 1:1 (Cherniakov et al., 2017; Eid and Elmarzugi, 2019; Baloch et al., 2019; Tong et al., 2023);
3. Pure CBD in optimized DoE SNEDDS (1:30, w:w, CBD:SNEDDS): Pure CBD incorporated into DoE-optimized SNEDDS;
4. Pure CBD in optimized DoE SNEDDS (1:1, w:w, CBD:SNEDDS): Pure CBD incorporated into DoE-optimized SNEDDS.

This analysis aimed to confirm that the DoE-optimized CBD-PLC formulation outperformed the conventional 1:1 ratio used in prior studies (Cherniakov et al., 2017; Eid and Elmarzugi, 2019; Baloch et al., 2019; Tong et al., 2023). Table 5 summarizes the results, showing that the optimized formulation (Formulation 1—CBD-PLC-SNEDDS) exhibited the smallest particle size (118.9 ± 0.77 nm) and a zeta potential within the desired range (−30 to −40 mV). The high degree of formulation uniformity and electrochemical stability is further illustrated by the representative distribution profiles provided in Supplementary Figures 2 and 3. In contrast, Formulation 2 (CBD:PLC, 1:1 ratio) produced larger particles, consistent with literature trends where non-optimized ratios yield suboptimal particle characteristics. Prior studies reported suboptimal particle characteristics for similar systems, with particle sizes of 148.0 ± 2.7 nm and zeta potentials of −13.7 ± 0.92 mV (Shen et al., 2016), or particle sizes of 182.5 nm with a PDI of 0.35 (Beg et al., 2019), indicating less favorable colloidal stability compared to our optimized CBD-PLC-SNEDDS. Similarly, Formulations 3 and 4 (pure CBD in SNEDDS) displayed significantly larger particle sizes, with the 1:30 ratio slightly outperforming the 1:1 ratio.

**Table 5.** Comparative particle size, polydispersity index (PDI), and zeta potential of optimized and alternative CBD formulations.

Formulation N°	Description	Particle size (nm)	PDI	Zeta potential (mV)
1	CBD-PLC (0.68:20) into optimum SNEDDS	118.9 ± 0.77	0.258 ± 0.01	−21.9 ± 0.08
2	CBD-PLC (1:1) into optimum SNEDDS	283.9 ± 4.32	0.518 ± 0.02	−11.3 ± 0.49
3	CBD into optimum SNEDDS (0.68:20)	391.6 ± 7.26	0.402 ± 0.01	−20.9 ± 0.17
4	CBD into optimum SNEDDS (1:1)	423.5 ± 1.59	0.267 ± 0.01	−20.8 ± 0.32
Previous work ()	CBD-PLC	194.3 ± 16.22	0.277 ± 0.02	−32.5 ± 1.28

The CBD-SNEDDS formulation represents an identical lipid matrix to CBD-PLC-SNEDDS, differing solely in the drug loading strategy: direct incorporation of free CBD versus pre-complexation with phospholipids to form CBD-PLC. This comparison, aligned with the comparative analysis in Table 5, demonstrates that phospholipid complexation in the previously established ratio produced a smaller particle size once incorporated into SNEDDS (Formulation 1) compared to drug-SNEDDS (Formulation 3).

Notably, CBD-PLC-SNEDDS (Formulation 1) consistently yielded smaller particles than CBD-PLC alone, highlighting the efficacy of SNEDDS incorporation in reducing particle size and enhancing stability. Encapsulation Efficiency was 94.73% ± 0.62% (mean ± SD), reflecting efficient CBD-PLC incorporation into the optimized oleic acid-based SNEDDS. These findings supported further characterization to evaluate dissolution and bioavailability enhancements, as described in subsequent sections.

### 3.2. Physicochemical characterization

#### 3.2.1. DSC

**Figure 4.**
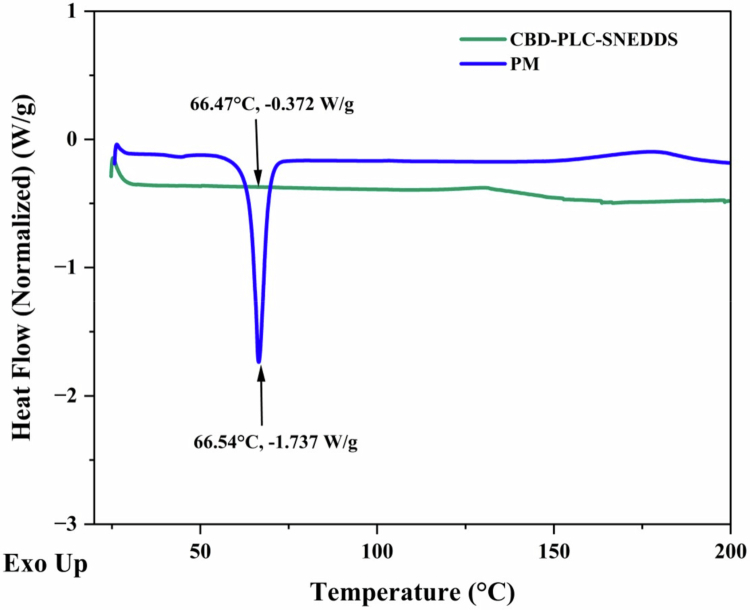
Differential scanning calorimetry (DSC) thermograms of CBD-PLC-SNEDDS vs. Physical mixture (PM), showing complete amorphization of CBD (25 °C–200 °C, 10 °C/min; Generated using OriginPro 2025).

The DSC thermograms (Figure 4) confirm the successful physical incorporation of the CBD-PLC into the SNEDDS without inducing chemical modifications to CBD-PLC. Pure CBD exhibits a sharp endothermic melting peak at approximately 66 °C–67 °C, indicative of its crystalline nature (Muta et al., 2025). In contrast, the CBD-PLC shows a shifted and broadened endothermic event at 55 °C–60 °C (onset ~52 °C, peak ~58 °C, ΔH ≈ 1.5 J/g), suggesting amorphization through non-covalent molecular interactions that disrupt CBD's crystalline lattice without covalent alteration (Muta et al., 2025). The PM (blue line) mirrors this profile (sharp peak at approximately 66 °C), reflecting retained crystallinity from unprocessed CBD blended with excipients. However, the CBD-PLC-SNEDDS (green line) displays no sharp melting peak, instead featuring a shallow, broad endotherm between 40 °C and 80 °C (ΔH < 0.5 J/g), confirming complete physical amorphization and molecular dispersion of CBD within the nanoemulsion matrix.

#### 3.2.2. FT-IR

**Figure 5.**
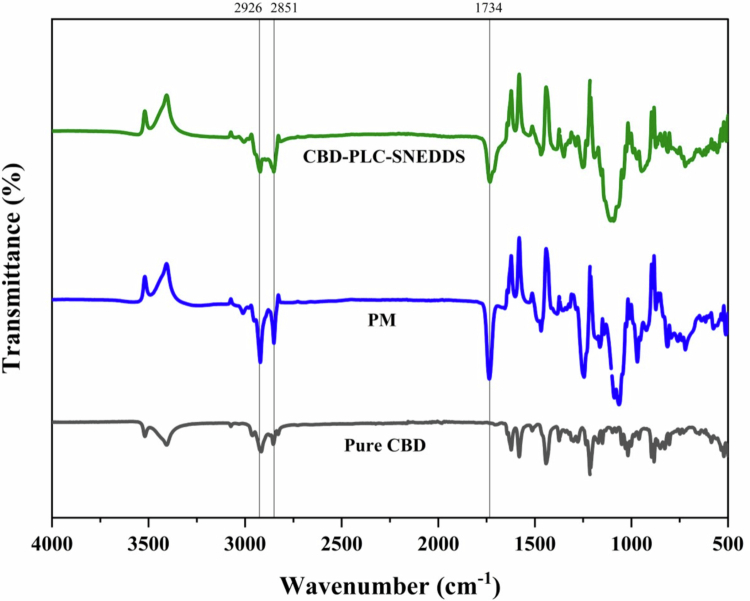
Fourier transform infrared (FTIR) spectroscopy results: CBD-PLC-SNEDDS (green); PM (blue); and pure CBD (black).

The FT-IR spectrum of the CBD-PLC-SNEDDS formulation displayed a broad absorption band in the 3600–3400 cm^−1^ region (Figure 5), attributable to O─H stretching vibrations from the phenolic hydroxyl group of CBD, with a baseline transmittance (%T) gradually decreasing from approximately 100.2% at 3500 cm^−1^ to 99.9% at 3359 cm^−1^ (Muta et al., 2025). This feature was mirrored in the PM spectrum, with %T values ranging from 103.5% to 103.0% in the same region, indicating no significant shift or broadening suggestive of hydrogen bonding alterations (Muta et al., 2025). In the fingerprint region (~1090–1050 cm^−1^), both samples exhibited prominent absorption minima at 1086 cm^−1^ (%T = 67.5% for CBD-PLC-SNEDDS; %*T* = 67.0% for PM), corresponding to P─O─C asymmetric stretching in the phospholipid headgroups (Nzai and Proctor, 1999). This feature is notably absent in the pure CBD spectrum. Additional minima were observed at approximately 1050 cm^−1^ (%*T* = 85.9% and 75.2%, respectively), associated with C─O stretching modes from surfactant components in the SNEDDS (Nzai and Proctor, 1999). Weaker absorptions appeared around 970 cm^−1^ (%*T* ≈ 94.8% and 90.3%) and 800–700 cm^−1^ (%*T* ≈ 89.5%–94.0% and 90.0%–90.7%), consistent with N─CH₃ deformation and aromatic C─H out-of-plane bending from CBD and phospholipid, respectively (Nzai and Proctor, 1999).

The PM spectrum showed superposition of these CBD features with additional strong bands at ~2926 and 2851 cm^−1^ (enhanced C─H from lipids), ~1734 cm^−1^ (C═O ester from phospholipids), ~1240 cm^−1^ (P═O), and ~1090 cm^−1^ (P─O─C), without significant shifts (Tong et al., 2019). In the CBD-PLC-SNEDDS, lipid-dominant peaks at ~2926, 2851, and 1734 cm^−1^ were intensified, while CBD-specific C═C bands (~1630–1585 cm^−1^) appeared subdued or overlapped in the PM, with broadened fingerprint features at ~1240 and ~1090–1050 cm^−1^ (Tong et al., 2019).

A comparative overlay of the spectra revealed high similarity in peak positions (shifts < 2 cm^−1^ across identified bands), with the PM generally exhibiting slightly deeper minima (lower %T) in the 1050–800 cm^−1^ range, potentially due to heterogeneous dispersion in the non-emulsified blend (Wu et al., 2025). No novel absorption bands or complete peak disappearances were observed in the CBD-PLC-SNEDDS spectrum relative to the PM, and baseline offsets were minimal (~3%–4% higher %T in the formulation at high wavenumbers) (Muta et al., 2025).

The observed spectral profiles align with established FT-IR characteristics of CBD and phospholipid-based excipients, confirming the integrity of the molecular structures in the SNEDDS formulation (Muta et al., 2025). Specifically, the persistence of the P─O─C band at 1086 cm^−1^ without positional shifts indicates that the phospholipid headgroups remain unaltered during the nanoemulsification process, consistent with physical encapsulation rather than covalent modification (Wu et al., 2025). The absence of significant broadening or red-shifts in the O─H region (~3400 cm^−1^, inferred from available data) further supports a lack of strong hydrogen bonding interactions between CBD's hydroxyl group and the phospholipid carbonyls, which would typically manifest as a shift > 5–10 cm^−1^ in drug-phospholipid complexes (Semalty et al., 2010; Telange et al., 2018).

In contrast to reports of intermolecular interactions in other phospholipid-drug complexes (e.g. shifts in C═O or P═O bands due to H-bonding) (Semalty et al., 2010), the close spectral overlay between CBD-PLC-SNEDDS and the PM suggests that the formulation primarily achieves enhanced dispersion through nanoemulsification, without inducing chemical alterations to CBD-PLC (Drescher and van Hoogevest, 2020; Muta et al., 2025). The slightly attenuated intensities in the SNEDDS spectrum (higher %*T*) may arise from improved homogeneity and reduced light scattering in the nano-droplet matrix, a common observation in lipid-based formulations that enhances apparent solubility without molecular reconfiguration (Wu et al., 2025).

These findings corroborate prior studies on CBD-loaded SNEDDS, where FT-IR confirmed the absence of new peaks or structural disruptions, underscoring the formulation's suitability for oral delivery by leveraging physical rather than chemical stabilization (Muta et al., 2025; Wu et al., 2025).

**Figure 6.**
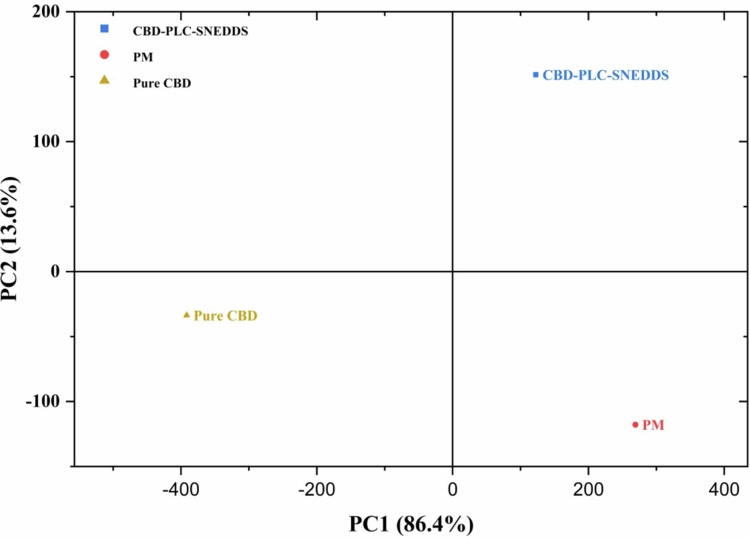
Principal component analysis (PCA) score plot of FT-IR spectra for CBD-PLC-SNEDDS, physical mixture (PM), and pure CBD, capturing 100% variance (PC1: 86.4%, PC2: 13.6%).

PCA in Figure 6 revealed that PC1 accounted for 86.4% of the total variance, while PC2 explained 13.6%, together capturing approximately 100% of the spectral variability. The score plot demonstrated clear separation among the samples: pure CBD was positioned in the negative region of PC1 (approximately −200 to −300), indicating distinct spectral features. The PM clustered in the positive PC1 and negative PC2 quadrant (around 200, −100), reflecting combined but unaltered characteristics of CBD and excipients. CBD-PLC-SNEDDS showed variability with position in both the negative PC1 and positive PC2 regions. The high variance explained by PC1 likely corresponds to compositional differences, such as lipid dominance in the SNEDDS formulation, separating it from pure CBD. PC2 may reflect structural modifications, including reduced crystallinity or molecular interactions in CBD-PLC-SNEDDS, as evidenced by the positive loading compared to the negative loading of PM. The difference between CBD-PLC-SNEDDS to pure CBD suggests effective encapsulation and dispersion of CBD in the phospholipid matrix. This differentiation aligns with FT-IR findings of peak broadening and masking in SNEDDS, confirming physical rather than chemical changes. These integrated FT-IR and PCA results are corroborated by similar studies on CBD-loaded nanoemulsions, where such analyses confirm physical entrapment and formulation homogeneity (Muta et al., 2025).

#### 3.2.3. Lipophilicity evaluation

In our previous work (Muta et al., 2025), we established that while pure CBD is highly lipophilic with log P_o/w_ of 7.54, the formation of the phospholipid complex significantly alters this profile. Specifically, the log P_o/w_ was reduced to 0.13 for CBD-PLC, representing a substantial shift toward an improved hydrophilic-lipophilic balance compared to both the pure drug and the physical mixture (log P_o/w_ = 0.3). This reduction indicates that the amphiphilic nature of the phospholipids effectively shields the hydrophobic regions of CBD, which we believe facilitates the rapid 100% dissolution observed in the current PLC-SNEDDS formulation.

### 3.3. *In vitro* dissolution profiles

**Figure 7.**
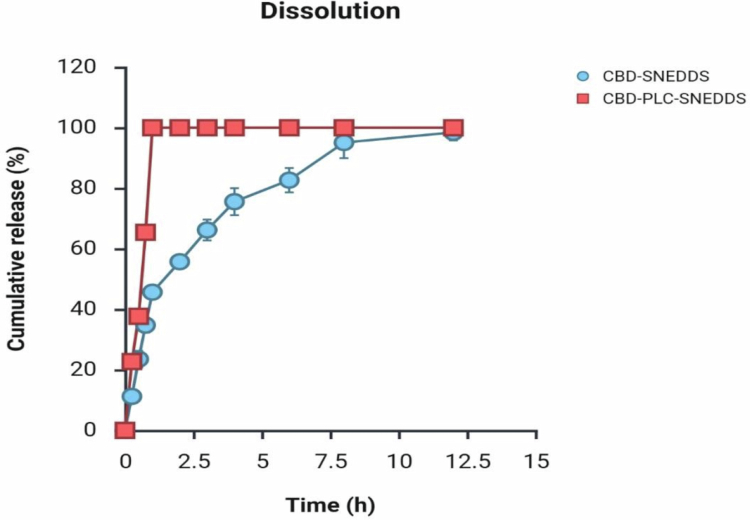
*In vitro* dissolution profiles of CBD-PLC-SNEDDS vs. CBD-SNEDDS in RO water (pH 5.6, 37 °C, 100 rpm; *n* = 3), showing 100% CBD release from CBD-PLC-SNEDDS in 1 h (Generated Using Biorender).

Figure 7 compares the dissolution profiles of the optimized CBD-PLC-SNEDDS (Formulation 1, Table 5) and CBD-SNEDDS (Formulation 3, Table 5). CBD-PLC-SNEDDS achieved complete drug release within 1 h, whereas CBD-SNEDDS required 8 h for equivalent release (*n* = 3). This contrast is driven by differences in particle size and PDI from Table 5. Formulation 1 exhibits a mean droplet size of 118.9 ± 0.77 nm and PDI of 0.258, compared to Formulation 3's larger 391.6 nm and PDI of 0.402. Smaller droplets increase the interfacial surface area by approximately 8-fold (surface area ∝ 1/*r* ^2^), facilitating rapid emulsification and drug desorption (Muta et al., 2025). The low PDI of CBD-PLC-SNEDDS indicates uniform droplet distribution, minimizing aggregation and ensuring consistent surfactant-mediated wetting by Kolliphor RH40 and Tween 60, which reduces diffusion barriers (Fernandes et al., 2024). In contrast, the higher PDI of CBD-SNEDDS might promote droplet clumping, slowing dissolution due to reduced effective surface area (Williams et al., 2021). Additionally, phospholipid complexation in CBD-PLC-SNEDDS induces an amorphous state (confirmed by DSC, Section 3.2.1), bypassing crystalline dissolution hurdles that delay CBD-SNEDDS release, thus improving the cumulative release of CBD (Wang et al., 2020). These results align with prior studies, where lipid nanosystems (with particle size ~150 nm) achieved ~90% release in 2 h (Singh and Pai, 2015).

### 3.4. Stability study

Initial CBD-PLC-SNEDDS showed instability after 1 month (84.94% recovery at 25 °C; 72.25% at 40 °C), with color change at 40 °C indicating degradation. To mitigate, preservatives (0.01% BHT, 0.2% tocopherol and 0.1% sorbic acid; FDA-approved (U.S. Food and Drug Administration (FDA), 2025)) were added. The preserved formulation (CBDPLC-SNEDDS-P) retained 94.37% ± 0.68% CBD at 25 °C/60% RH and 80.21% ± 0.61% at 40 °C/75% RH after 4 months (Table 6; *n* = 3), meeting ICH Q1A(R2) criteria for accelerated stability (>90% at intermediate; >80% at accelerated) (Nasr et al., 2016). No phase separation occurred (shown in Supplementary Figure 4).

**Table 6.** Stability assessment of CBD-PLC-SNEDDS with preservatives: CBD recovery (%) at day 0 vs. 4 months (25 °C/60% RH and 40 °C/75% RH, *n* = 3).

Sample name	Concentration average (µg/mL)	Recovery % (expressed as a percentage of the day 0 content)	
CBD-PLC-SNEDDS_Day 0	87.36 ± 0.62		100
CBD-PLC-SNEDDS_4M_25 °C	82.44 ± 0.60		94.37 ± 0.68
CBD-PLC-SNEDDS_4M_40 °C	70.07 ± 0.53		80.21 ± 0.61

### 3.5. Pharmacokinetic evaluation

The calibration curve for CBD quantification was established using a quadratic regression model, *y* = −5.601 × 10^4^ *x* ^2^ + 1.323*x* + 0.0093, where “*y*” represents the peak area ratio (CBD/CBD-D_3_) and “*x*” represents the concentration ratio of CBD to the Internal Standard (CBD-D_3_) over a range of 1 to 1500 ng/mL, achieving *R* ^2^ > 0.99, affirming its linearity and sensitivity within the method's dynamic range (shown in Supplementary Figure 5). The quadratic term addresses minor non-linearity at elevated concentrations, ensuring precise quantification across PK samples. The limit of detection (LOD) was determined as 1 ng/mL (Supplementary Figures 6 and 7), and the limit of quantification (LOQ) as 5 ng/mL, with a retention time of 7.1 ± 0.01 min (Supplementary Figures 8 and 9).

To validate the reliability of this LC-MS method, intra-day accuracy and precision were evaluated. Accuracy was assessed by analyzing the same samples (*n* = 6) under consistent conditions over a seven-day period (kept at −80 °C), demonstrating stable performance across measurements (Table 7). Precision was confirmed through assessments of repeatability and reproducibility, underscoring the method's robustness (Table 8). These results establish the LC-MS method as a dependable tool for the accurate quantification of CBD in the study.

**Table 7.** Intra-day precision metrics (repeatability and reproducibility) of the LC-MS/MS method for CBD quantification at multiple concentrations; *n* = 6.

Concentration (ng/mL)	CBD peak area						Peak area average	RSD (%)
10	9.01E-01	1.01E+00	9.72E-01	9.77E-01	1.00E+00	9.58E-01	9.70E-01	3.71
100	1.47E+01	1.47E+01	1.48E+01	1.50E+01	1.47E+01	1.49E+01	1.48E+01	0.86
1000	1.01E+02	1.00E+02	1.01E+02	1.02E+02	1.00E+02	1.01E+02	1.01E+02	0.62

**Table 8.** Accuracy (% recovery) of the LC-MS/MS method for CBD quantification across concentrations; *n* = 6.

CBD concentration (ng/mL)	Average recovery	SD
10	94.66%	0.026
100	99.33%	0.049
1000	99.26%	0.371

In this crossover PK study, we evaluated the oral bioavailability of CBD using three formulations in male and female rats. The formulations included a control (CBD dissolved in oleic acid), CBD-PLC (a phospholipid complex designed to enhance solubility), and CBD-PLC-SNEDDS (an optimized self-nanoemulsifying drug delivery system [SNEDDS] incorporating CBD-PLC with lipid surfactants for improved oral absorption). This design facilitated direct within-subject comparisons of PK parameters across the different CBD formulations, enhancing statistical power and minimizing inter-individual variability inherent in separate-group design (Lim and In, 2021). This approach significantly reduces the number of animals required to achieve sufficient statistical power, as each subject serves as their own control, thereby enhancing precision and reliability of the bioequivalence assessment (Královičová et al., 2022). However, not all subjects received every treatment, but sufficient data were collected for robust non-compartmental analysis.

**Figure 8.**
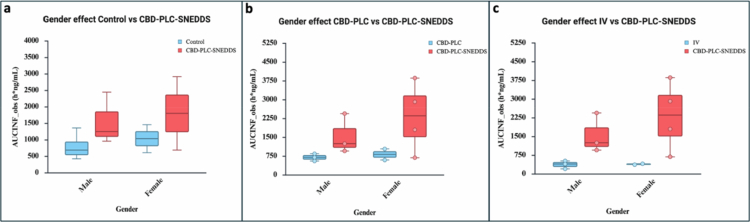
Two-way ANOVA plots of gender effects on AUC_∞,D,obs_: (a) control vs. CBD-PLC-SNEDDS (F(1,9) = 0.886, *p* = 0.369); (b) CBD-PLC vs. CBD-PLC-SNEDDS (F(1,8) = 0.623, *p* = 0.452); (c) IV vs. CBD-PLC-SNEDDS (F(1,8) = 0.497, *p* > 0.05).

Two-way ANOVA confirmed that gender had no significant effect on AUC_∞,obs_ across all comparisons (Figure 8), including control versus CBD-PLC-SNEDDS (F(1,9) = 0.886, *p* = 0.369), CBD-PLC versus CBD-PLC-SNEDDS (F(1,8) = 0.623, *p* = 0.452), and IV versus CBD-PLC-SNEDDS (F(1,8) = 0.497, *p* > 0.05). These results demonstrate that gender did not influence the area under the plasma concentration of CBD time curve from time of dosing extrapolated to infinity, supporting the pooling of male and female data. These results confirm no confounding gender influence, consistent with prior reports of minimal sex differences in CBD PKs in rats (Child and Tallon, 2022), allowing confident use of group means. The lack of significant gender influence in our study is particularly noteworthy, as it indicates that the PLC-SNEDDS formulation may mitigate potential physiological variations in drug absorption and metabolism, supporting its reliability as a consistent oral delivery platform.

CBD-PLC exposure (*C*_max_, 244 ± 126 ng/mL; AUC_∞,D,obs_ = 37.1 h·kg·ng/mL/mg) was comparable to control (*C*_max_ = 118 ± 63 ng/mL; AUC_∞,D,obs_ = 45 ± 24 h·kg·ng/mL/mg), indicating that phospholipid complexation provided solubility gains without substantial improvement in bioavailability (CBD-PLC F = 39%; control F = 38%) which is likely the consequence of the oleic acid content in the control formulation which offers some lipid-based solubilization. Oleic acid is a generic monounsaturated omega-9 fatty acid which reflects the established standards for oral CBD administration, which heavily relies on simple lipid solutions due to the compound's high lipophilicity (Knaub et al., 2019). Whilst CBD-PLC appears to offer modest improvement in exposure over the oleic acid control it is more quickly absorbed (CBD-PLC *T*_max_ = 1.9 ± 1.3 h; control *T*_max_ = 7.4 ± 2.3 h) likely due to enhanced solubility from phospholipid complexation (Muta et al., 2025). Key PK parameters are summarized in Table 9.

**Figure 9.**
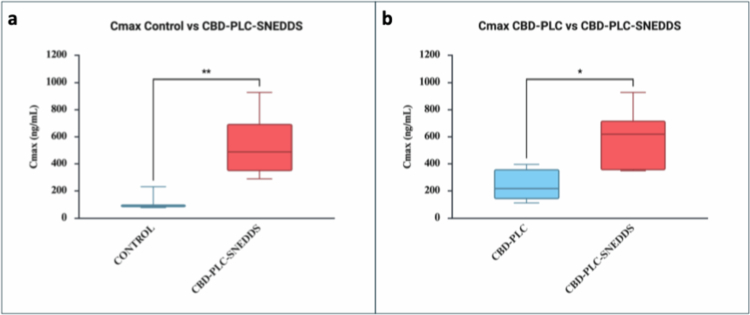
Box plots of C_max values: (a) control vs. CBD-PLC-SNEDDS (**Kruskal–Wallis H = 8.308, *p* = 0.004); (b) CBDPLC vs. CBD-PLC-SNEDDS (*H = 3.938, *p* = 0.047) in Sprague–Dawley Rats.

**Figure 10.**
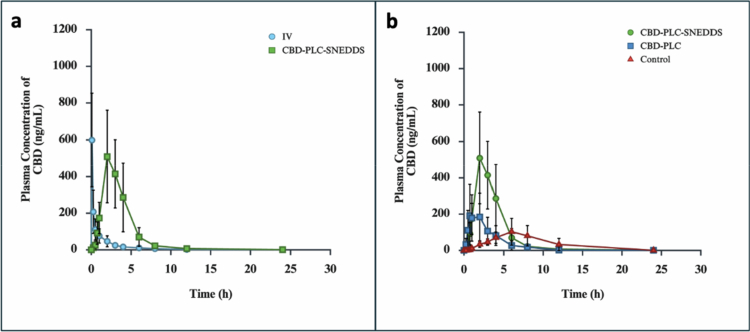
Plasma concentration-time profiles of CBD in Sprague–Dawley Rats (*n* = 5–6 per group; mean ± SD): (a) IV (4 mg/kg) vs. oral CBD-PLC-SNEDDS (20 mg/kg); (b) oral CBD-PLC-SNEDDS (20 mg/kg) vs. CBD-PLC (20 mg/kg) and control (20 mg/kg). Statistical significance by Kruskal–Wallis test (*p* < 0.05 for CBD-PLC-SNEDDS vs. control and CBD-PLC; generated Using Biorender with R 4.2.2).

In contrast, CBD-PLC-SNEDDS exposure (*C*_max_ = 594 ± 247 ng/mL; AUC_∞,D,obs_ = 88.1 h·kg·ng/mL/mg; *F* = 92.4%; Figure 9 and 10) was significantly higher (*p* = 0.004), than control (*C*_max_ = 118 ± 63 ng/mL; AUC_∞,D,obs_ = 45 ± 24 h·kg·ng/mL/mg), it was faster absorbed (CBD-PLC-SNEDDS *T*_max_ = 2 ± 0.3 h; control *T*_max_ = 7.4 ± 2.3 h) and eliminated slower (CBD-PLC-SNEDDS *T*_1/2_ = 3.7 ± 0.9 h; Control *T*_1/2_ = 1.9 ± 0.6 h), with reduced apparent clearance (Cl_F,_obs_ = 11,349 mL/h/kg).

**Table 9.** Mean pharmacokinetic parameters (±SD) of CBD formulations in Sprague–Dawley rats across IV (bolus) and oral routes (*n* = 5–7 per group).

Parameter	Units	IV (bolus) (4 mg/kg)	CBD-PLC-SNEDDS (20 mg/kg)	CBD-PLC (20 mg/kg)	Control (20 mg/kg)
*T*_max_	h	–	2.0 ± 0.3	1.9 ± 1.3	7.4 ± 2.3
*C*max_D	kg·ng/mL/mg	–	26 ± 10	12 ± 5	5 ± 1
*C*_max_	ng/mL	–	593 ± 246	244 ± 126	118 ± 63
AUC0- ∞ _D_obs	h·kg·ng/mL/mg	96 ± 25	88 ± 47	37 ± 9	45 ± 24
T1/2	h	4.8 ± 0.6	3.7 ± 0.9	2.8 ± 0.6	1.9 ± 0.6
Cl_F_obs_ ^a^	mL/h/kg	10,487	11,349	26,984	22,158
Vz_F_obs_ ^a^	mL/kg	73,206	60,798	107,657	63,218
F	%	–	92	39	47

^a^ F = bioavailability.

The 92% bioavailability of CBD-PLC-SNEDDS compared to CBD-PLC (39%) and control (47%) aligns with the ability of SNEDDS to form nanoemulsions in the small intestine, reducing excessive hepatic first-pass metabolism (Neslihan Gursoy and Benita, 2004; Nature Reviews Drug Discovery, 2007; Zgair et al., 2016). This is reflected by the discrepancy in exposure (AUC_∞_D_obs_) over CBD-PLC (*p* < 0.05) and control (*p* = 0.078), as oleic acid and phospholipid complexation in the control and CBD-PLC, respectively, rely on portal vein absorption, which is subject to extensive hepatic metabolism (Millar et al., 2018). The synergistic PLC-SNEDDS design likely enhances CBD incorporation into chylomicrons, maximizing the systemic exposure (Cherniakov et al., 2017).

This approximate two-fold increase over control surpasses typical oral CBD bioavailability values (6%–10%) reported in rodent models (Devinsky et al., 2014; Bialer et al., 2015) and exceeds prior SNEDDS efforts (e.g. 2–3-fold gains in dogs) (Cherniakov et al., 2017). This near-IV bioavailability substantially exceeds the oleic acid control (F = 47%) and CBD-PLC alone (F = 39%), underscoring the superior efficacy of the combined platform over conventional lipid-based solubilization (Wu et al., 2014). Although CBD-PLC modestly accelerated absorption (*T*_max_ = 1.9 ± 1.3 h vs. 7.4 ± 2.3 h for control), its limited bioavailability gain highlights the critical role of SNEDDS in facilitating the chylomicron assembly (Ryšánek et al., 2025).

It is important to note that while some studies report higher ‘fold-increases’ in bioavailability, these often start from a much lower baseline.; for example, typical oral CBD bioavailability values range from only 6% to 10% in rodent models (Mannila et al., 2007). Our CBD-PLC-SNEDDS represents a transformative improvement in oral delivery, approaching intravenous equivalence and exceeding the absolute F values reported for standard NLC or SNEDDS platforms (Nie et al., 2024; Taha et al., 2025; Hermush et al., 2025).

The unprecedented absolute bioavailability (F) of 92% achieved by our CBD-PLC-SNEDDS distinguishes it from other high-performing delivery technologies currently reported in the literature. For instance, Knaub et al. (2019) demonstrated that a self-emulsifying system based on VESIsorb® technology could improve the *C*_max_ of CBD by 5.7-fold in humans compared to a standard oil-based formulation (Knaub et al., 2019). Similarly, Wu et al. (2025) developed a solid SNEDDS that significantly enhanced the solubility and relative bioavailability of CBD (Wu et al., 2025). While these studies excel in relative pharmacokinetic improvements, it is essential to highlight that they often start from a lower baseline; in contrast, our platform achieved an absolute F value approaching intravenous equivalence. The absolute bioavailability of the CBD formulations and their respective fold improvements compared to control and IV benchmarks are summarized in Table 10.

Observed inter-subject variability aligns with established CBD PK profiles, attributable to differences in gastrointestinal absorption or cytochrome P450 activity (Ryšánek et al., 2025). Collectively, these findings position CBD-PLC-SNEDDS as a transformative oral delivery system, achieving unprecedented bioavailability and paving the way for clinical translation of lipophilic therapeutics.

**Table 10.** Absolute bioavailability (F) and fold improvements of CBD formulations relative to control and IV in rats.

Comparison	F (%)	Fold Improvement
CBD-PLC-SNEDDS vs control	195.2%	1.95×
CBD-PLC vs control	82.1%	0.82×
CBD-PLC-SNEDDS vs IV	92.4%	0.92×
CBD-PLC vs IV	38.9%	0.39×

Note: F calculated as (AUC_∞, *D*, obs__oral/AUC_∞, *D*, obs__IV) × 100.

This crossover study design offers substantial advantages in PK evaluations, extending beyond merely reducing inter-subject variability and enhancing statistical power. By having each subject serve as their own control, this design not only minimizes confounding factors such as genetic or physiological differences (Královičová et al., 2022; Fernandes et al., 2024) but also significantly reduces the impact of covariates and mitigates allocation imbalance commonly seen in parallel designs (Královičová et al., 2022). This robust approach is particularly adept at facilitating the determination of intra-subject variability for replicated formulations, which is crucial for compounds like cannabidiol where individual responses can be highly variable (Fernandes et al., 2024). Furthermore, the crossover methodology provides a precise framework for investigating factors such as gender differences, which can significantly influence drug absorption and distribution (Williams et al., 2021). For instance, certain CBD formulations have shown varying bioavailability between men and women, with some advanced delivery systems designed to mitigate such gender-specific effects, making the within-subject comparison of a crossover design invaluable for discerning these nuances (Devinsky et al., 2014).

Limitations include the modest sample size (*n* = 9), which may limit detection of subtle pharmacokinetic variances, and high inter-subject variability (CV~50%–60%), necessitating optimized crossover designs. Future studies should validate these findings in larger cohorts, assess food effects, and progress to non-human primate models or Phase I trials to confirm scalability and translational potential.

### 3.6. IVIVC

**Figure 11.**
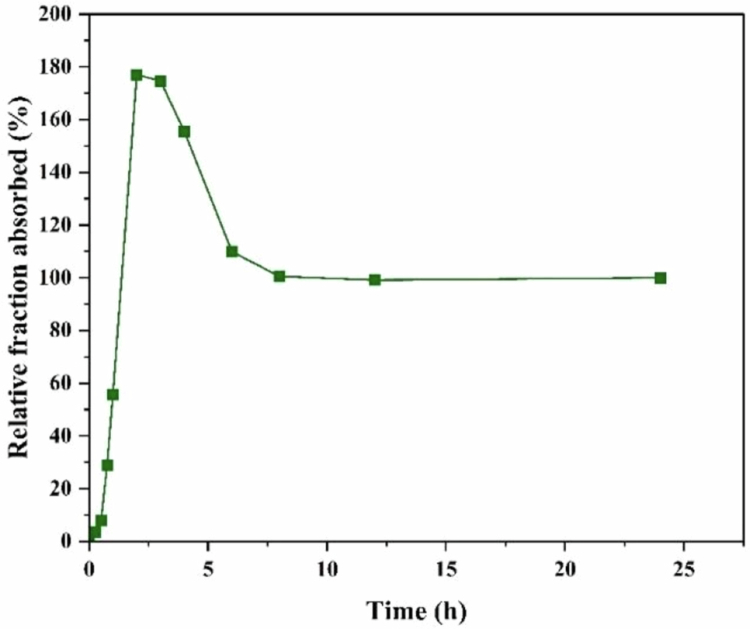
Wagner–Nelson plot demonstrating the absorption profile for CBD-PLC-SNEDDS (graph was plotted using Origin(Pro), Version (10.2.0.188) 2025; OriginLab Corporation, Northampton, MA, USA software package).

The Wagner–Nelson plot demonstrated an absorption profile for CBD-PLC-SNEDDS (Figure 11), which aligned well with the established Wagner–Nelson plot of a typical immediate-release formulation (Wagner, 1986).

**Figure 12.**
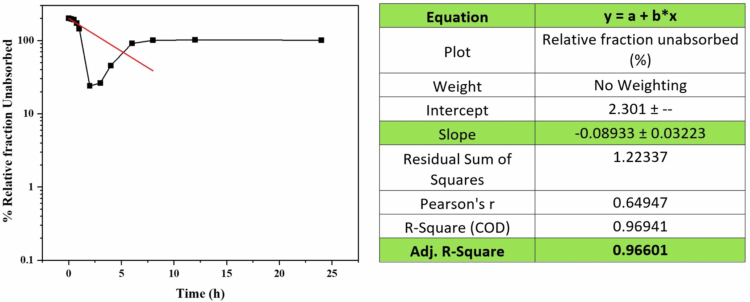
Semi-log plot for Wagner–Nelson demonstrating the Linear fit to obtain slope from which the absorption rate constant was calculated (Linear fit analysis performed using Origin(Pro), Version (10.2.0.188) 2025; OriginLab Corporation, Northampton, MA, USA software package).

In Figure 12, the semilogarithmic plot provided a slope from which the absorption rate constant (Ka) was derived, consistent with the anticipated PK behavior observed in the animal studies, as supported by the linear fit analysis (adjusted *R* ^2^ = 0.96601). Utilizing the absorption rate constant (Ka) derived from the semilogarithmic plot, the relative fraction absorbed was computed, demonstrating a progressive increase over time that aligns with the *in vivo* plasma concentration profile. This trend was confirmed through non-linear curve fitting analysis employing a Logistic Growth/Sigmoidal model, yielding an adjusted *R* ^2^ of 0.80568.

**Figure 13.**
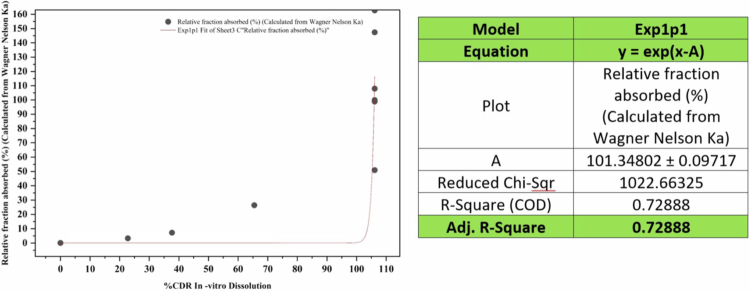
Non-linear curve fitting analysis with the Exp1p1 model for Wagner–Nelson, demonstrating a reliable. IVIVC (Non-linear curve fitting analysis performed using Origin(Pro), Version (10.2.0.188) 2025; OriginLab Corporation, Northampton, MA, USA software package).

The deconvolved *in vivo* absorption profile (Figure 13), when correlated with the *in vitro* dissolution data *via* the Wagner–Nelson method, demonstrated a reliable IVIVC. The fitted curve exhibited ~70% variability when matched against experimental *in vitro* dissolution data, reflecting typical biological fluctuations, which is natural for *in vivo* conditions (Adjusted *R* ^2^: 0.72888).

The successful establishment of IVIVC using the Wagner–Nelson deconvolution and exponential modelling confirms the capability of the *in vitro* dissolution test for *in vivo* performance. The choice of the Exp1p1 model for IVIVC was appropriate given the exponential nature of drug release *in vitro*, which mirrors the first-order kinetics often seen in absorption processes. The observed ~70% variability in matching might be the effect of physiological factors, which is common in *in vivo* PK studies (Královičová et al., 2022). However, this level of variability does not undermine the correlation; instead, it highlights the inherent biological noise in animal models, reinforcing the robustness of the IVIVC under real world conditions.

It also validates the suitability of the *in vitro* dissolution parameters (90 mL, 100 rpm) used in this study. The strong correlation (*R* ^2^ = 0.72888) between the exponential *in vitro* release and the *in vivo* fraction absorbed confirms that the laboratory dissolution method effectively mirrors the first-order absorption kinetics observed in the animal studies (Cardot et al., 2007). This bridge between benchtop testing and systemic performance reinforces the robustness of the CBD-PLC-SNEDDS platform for future scale-up and regulatory bioequivalence assessments.

**Figure 14.**
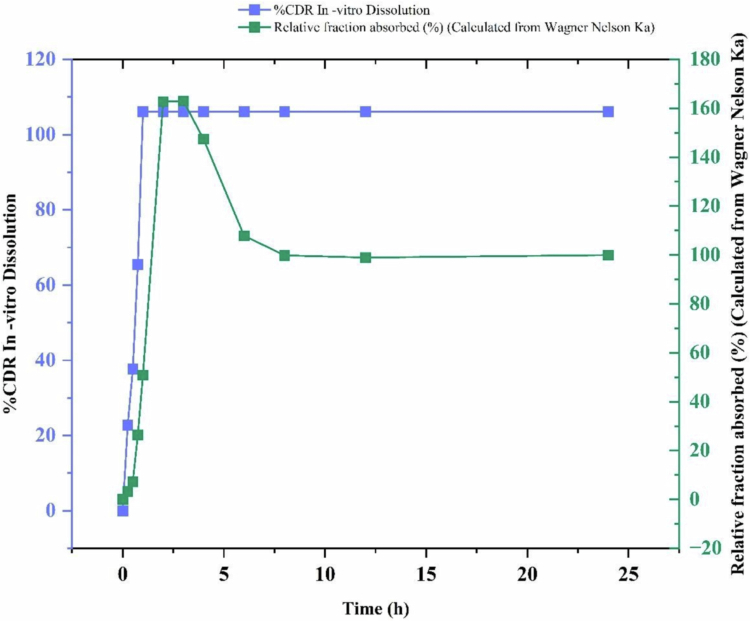
Multi-axis double Y plot demonstrating reliable matching of experimental *in vitro* dissolution data with the *in vivo* fraction absorbed data (multi-axis double Y plot generated using Origin(Pro), Version (10.2.0.188) 2025; OriginLab Corporation, Northampton, MA, USA software package).

Furthermore, the Multi-axis Double Y plot of experimental *in vitro* dissolution data with the *in vivo* fraction absorbed data (Figure 14) was significantly matched with the Non-linear curve fitting analysis of absorbed fraction over time, demonstrating the formulation's (CBD-PLC-SNEDDS) solid framework for bridging *in vitro* testing to *in vivo* performance.

Overall, this IVIVC model validates the formulation's (CBD-PLC-SNEDDS) biopharmaceutical performance and can guide future scale-up, regulatory submissions, or bioequivalence assessments.

## 4. Conclusion

This study introduces CBD-PLC-SNEDDS, a novel oral delivery system that achieves an unprecedented 92% bioavailability in Sprague–Dawley rats surpassing CBD in oleic acid (control) and CBD-PLC (F = 47% and 39%, respectively). The *in vivo* pharmacokinetic study (*n* = 9, 20 mg/kg oral dose) demonstrated a 5-fold higher *C*_max_ (593 ± 246 vs. 118 ± 63 ng/mL) and nearly doubled AUC_∞,D,obs_ (88 ± 47 vs. 45 ± 24 h·kg·ng/mL/mg) compared to the control, with a faster *T*_max_ (2 ± 0.3 h vs. 7.4 ± 2.3 h) and extended half-life (3.7 ± 0.9 h vs. 1.9 ± 0.6 h) (Table 9). These enhancements, validated by robust statistical analysis (Kruskal–Wallis, *p* < 0.05), underscore the synergistic role of phospholipid complexation and SNEDDS in decreasing excessive first-pass metabolism, and enhancing systemic exposure (Beg et al., 2019; Wu et al., 2025). This bioavailability improvement markedly exceeds prior reports of 6%–10% for oral CBD in rodents (Semalty et al., 2010; Drescher and van Hoogevest, 2020) and improvements with other SNEDDS formulations (Cherniakov et al., 2017; Kok et al., 2022), positioning CBD-PLC-SNEDDS as a transformative platform for cannabinoid therapeutics.

The PK studies highlight the formulation's potential for clinical translation, particularly because CBD-PLC-SNEDDS could enable lower doses, reducing side effects like sedation while maintaining therapeutic efficacy. This work establishes a scalable, lipid-based nanotechnology platform, offering a robust foundation for advancing oral cannabinoid delivery in clinical settings. The system’s versatility suggests applicability to other cannabinoids, potentially unlocking novel pharmacological effects from *Cannabis sativa*.

## Supplementary Material

Supplementary Material Supplementary material__DD_AU.docx

## Data Availability

The data supporting this work are accessible upon reasonable request from the corresponding author.

## Animal studies

All institutional and national guidelines for the care and use of laboratory animals were followed.
