# Supplementary material for: Development and in vivo pharmacokinetic evaluation of a phospholipid complex self-nanoemulsifying drug delivery system (PLC-SNEDDS) for enhanced oral bioavailability of cannabidiol
Source: Drug Deliv. 2026 Jul 22;33(1):2702143. doi: 10.1080/10717544.2026.2702143 (PMC13398106; doi:10.1080/10717544.2026.2702143)
Supplement: Supplementary Material — __DD_AU.docx [file IDRD_A_2702143_SM6303.docx]

**Development and *In Vivo* Pharmacokinetic Evaluation of a Phospholipid Complex Self-Nanoemulsifying Drug Delivery System (PLC-SNEDDS) for Enhanced Oral Bioavailability of Cannabidiol**

Thabata Muta^1^, Songhita Mukhopadhyay^1^, Benjamin Noll^1^, Yunmei Song^1^, and Sanjay Garg^1^.

^1^Adelaide University, Adelaide, SA, 5000, Australia

Corresponding author: [Sanjay.Garg@unisa.edu.au](mailto:Sanjay.Garg@unisa.edu.au)

**Supplementary Table S1.** Crossover study design

| **ID** | **Female-1** | | **Female-2** | **Female-3** | **Female-4** | **Male-1** | **Male-2** | **Male-3** | **Male-4** | **Male-5** |
| --- | --- | --- | --- | --- | --- | --- | --- | --- | --- | --- |
| **Dose 1** | Oral A | Control | | IV | Oral B | Oral A | Oral B | IV | Control | IV |
| **Dose 2** | Control | IV | | Oral B | Oral A | Control | Oral A | Oral B | IV | Control |
| **Dose 3** | Oral B | Oral A | | Control | IV | Oral B | IV | Control | Oral A | - |


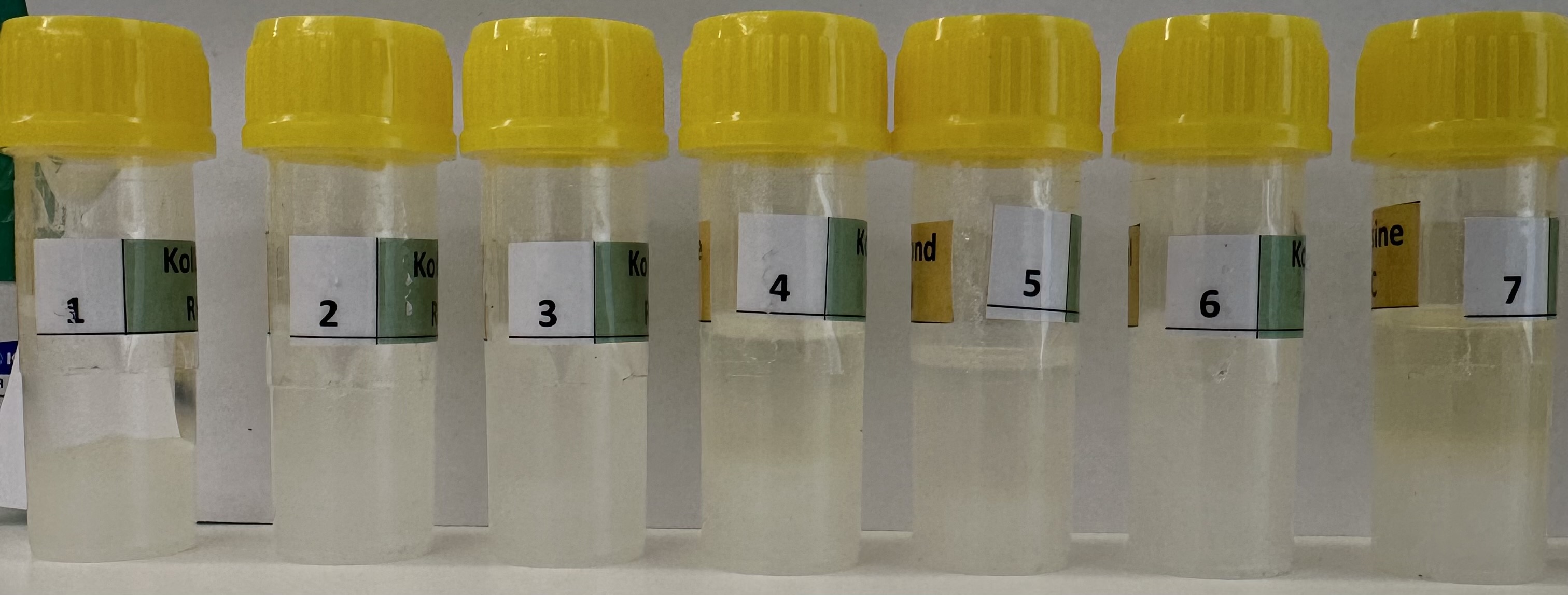


**Day 0**


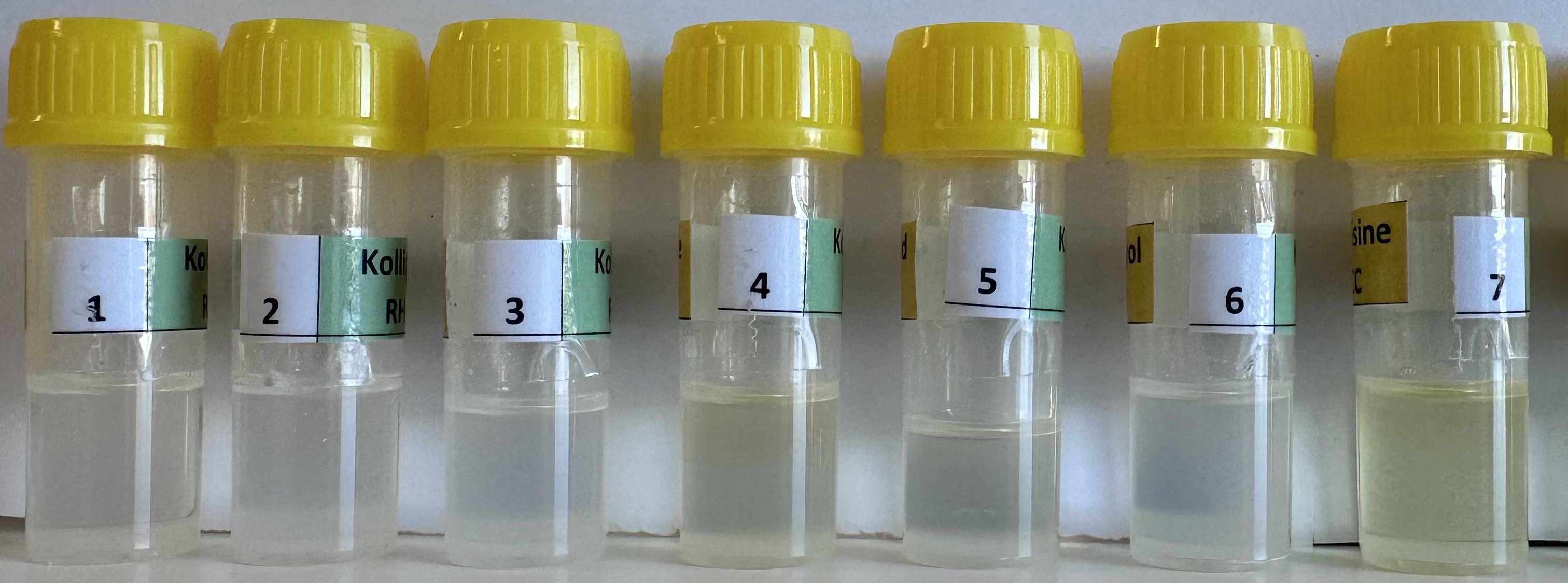


**Day 14**

**Day 0**


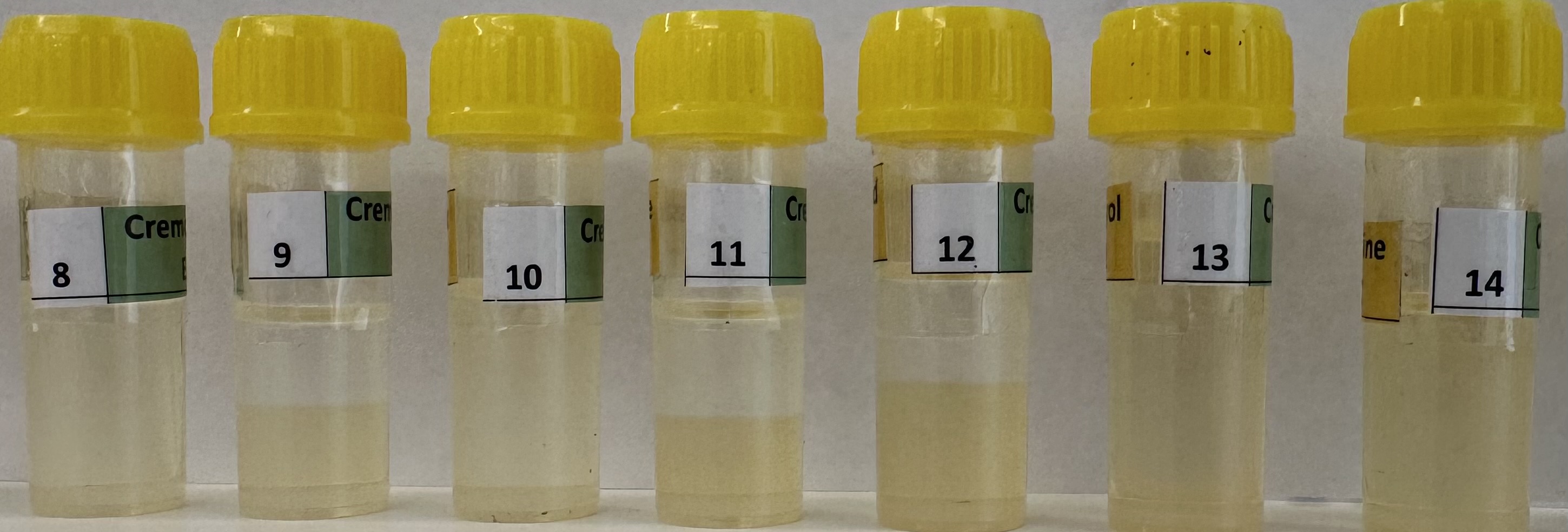


**Day 0**


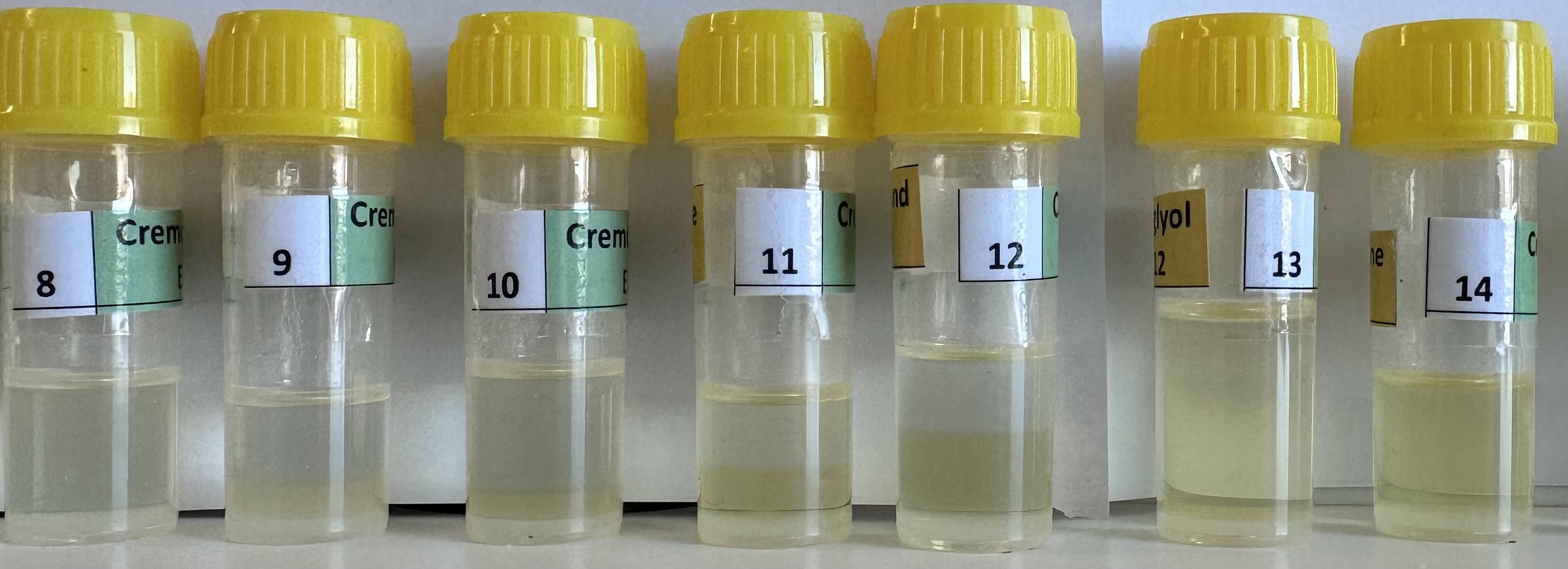


**Day 14**

**Day 0**


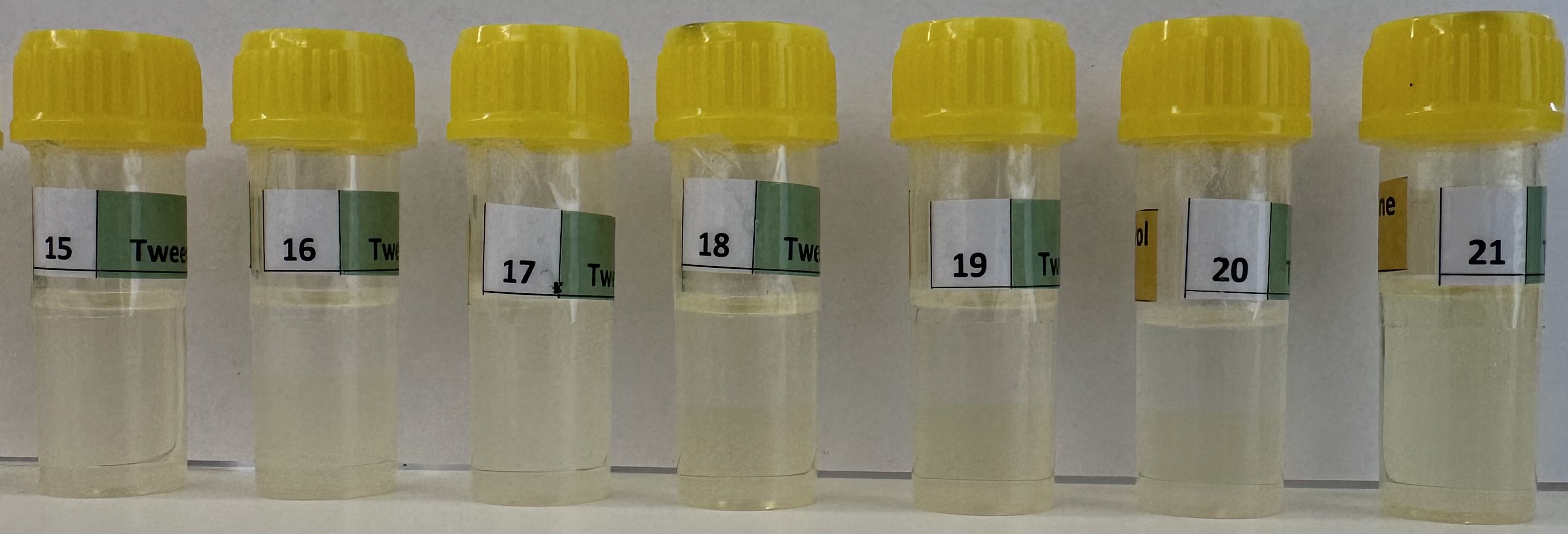


**Day 0**


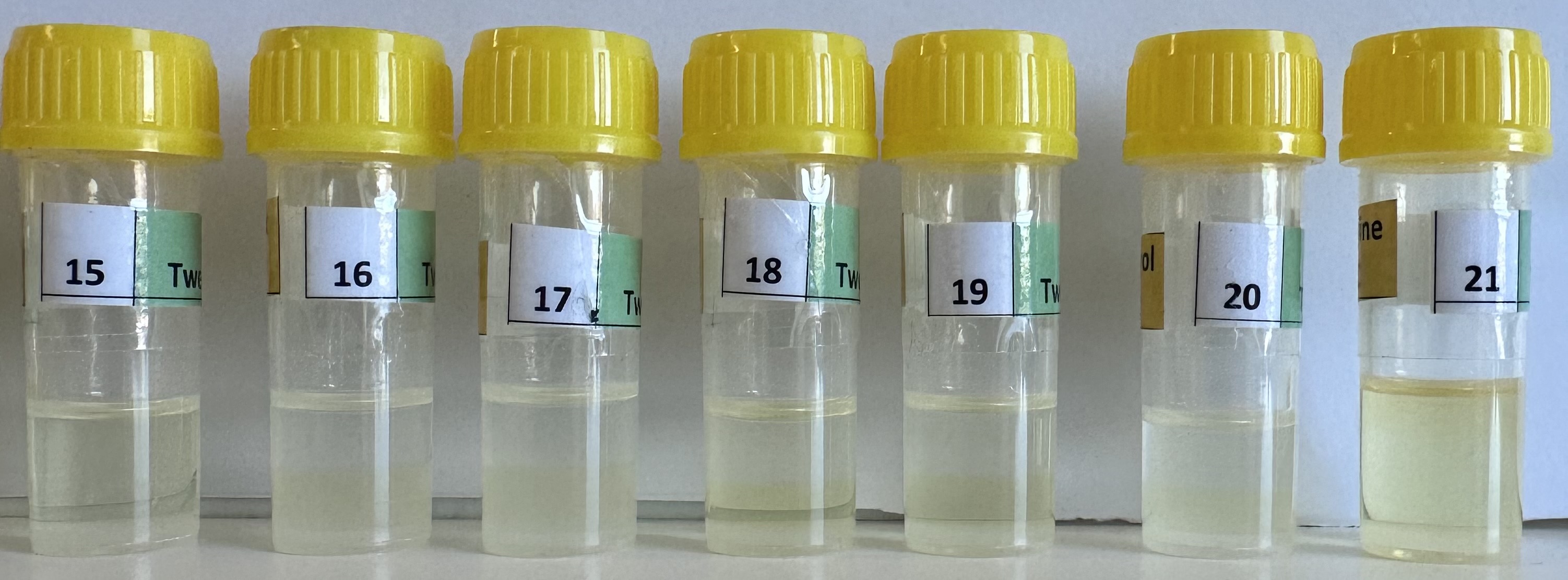


**Day 14**

**Day 0**


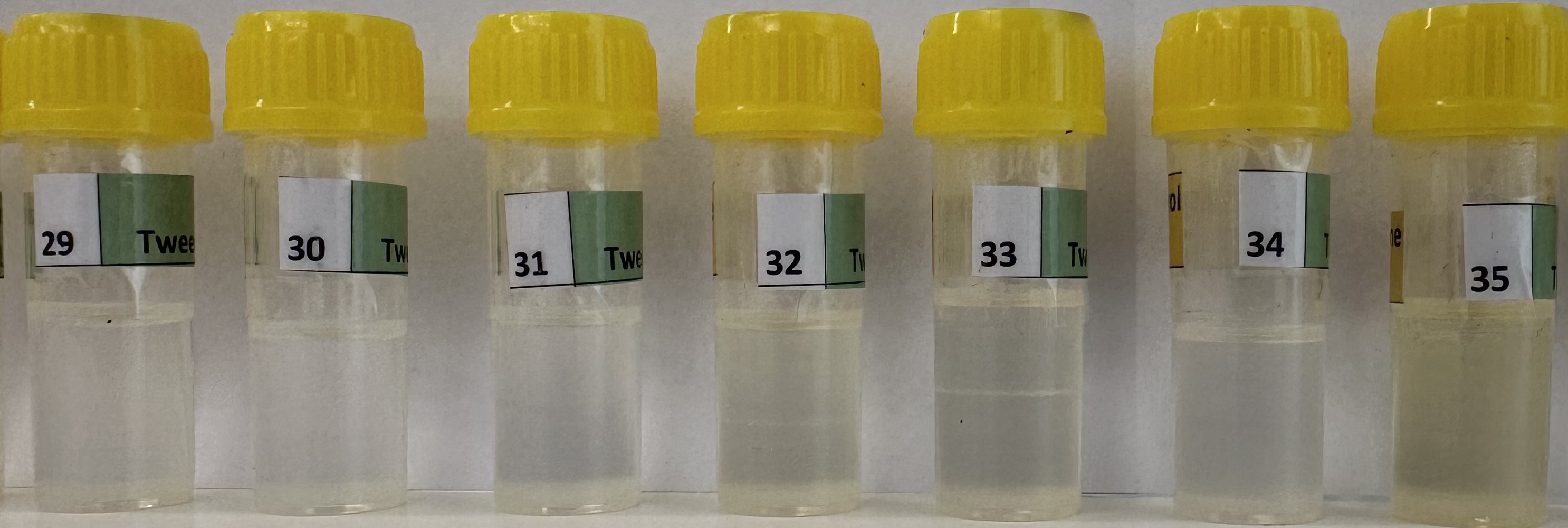


**Day 0**


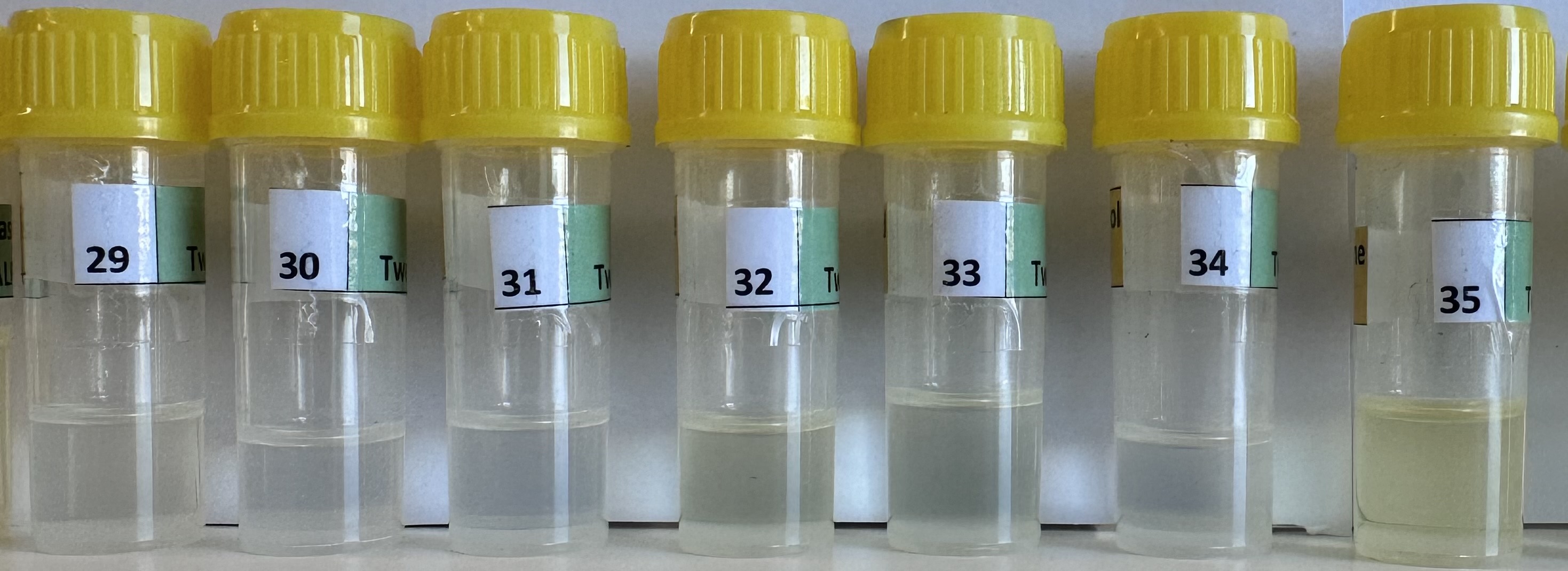


**Day 14**

**Day 0**


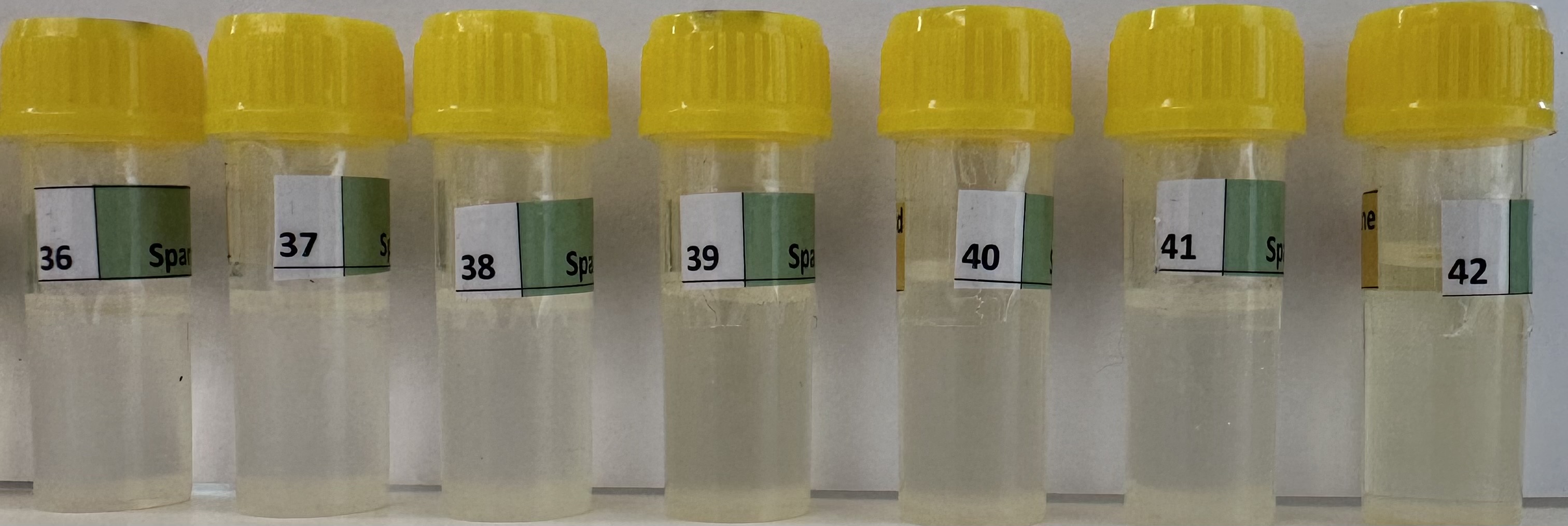


**Day 0**


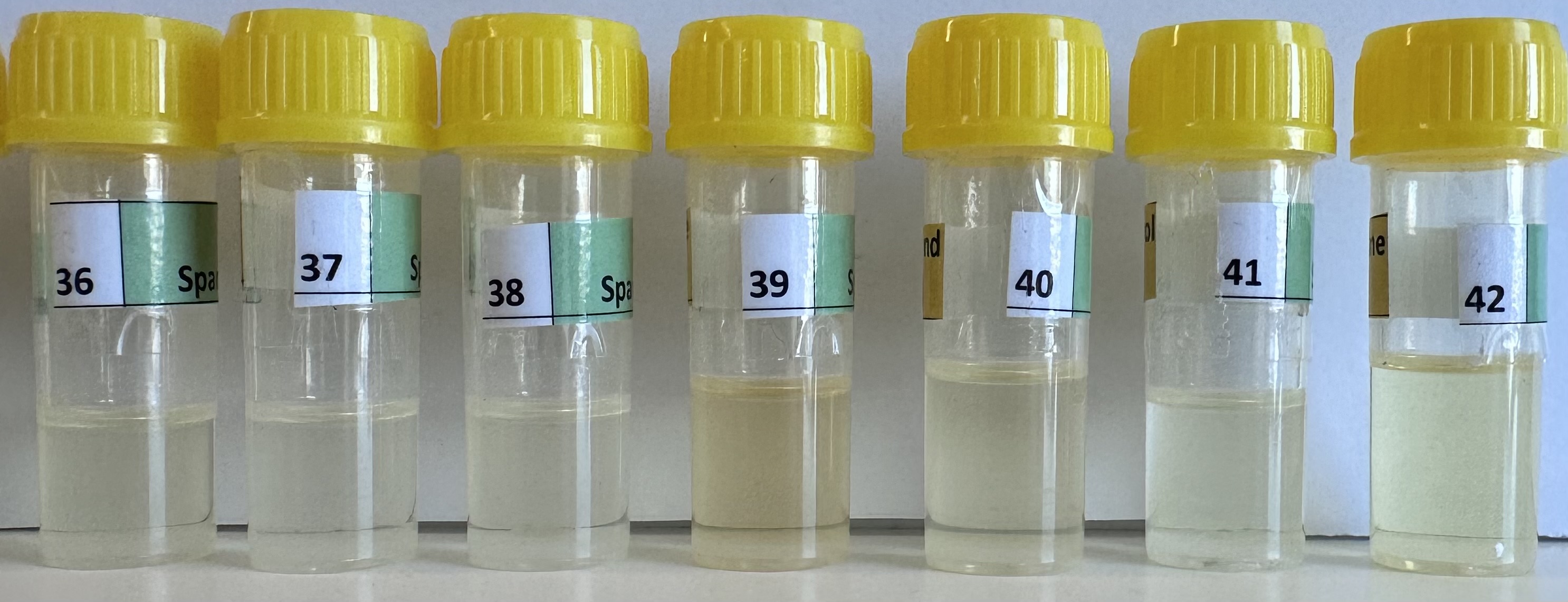


**Day 14**

**Day 0**


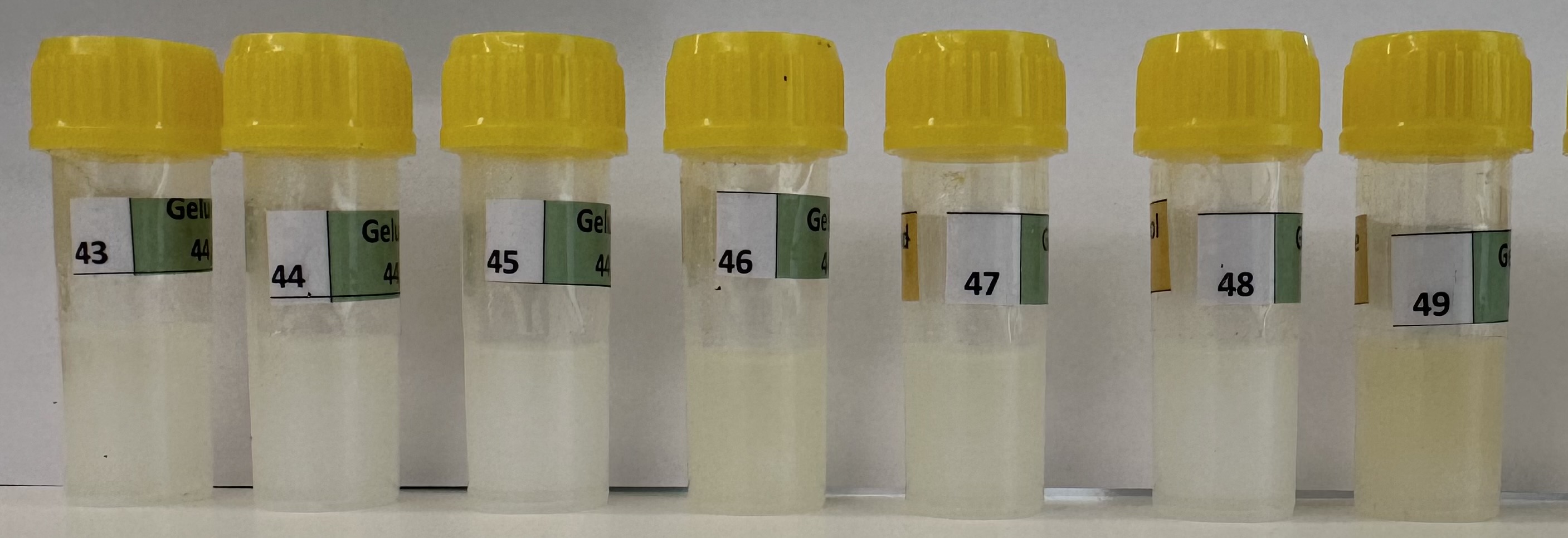


**Day 0**


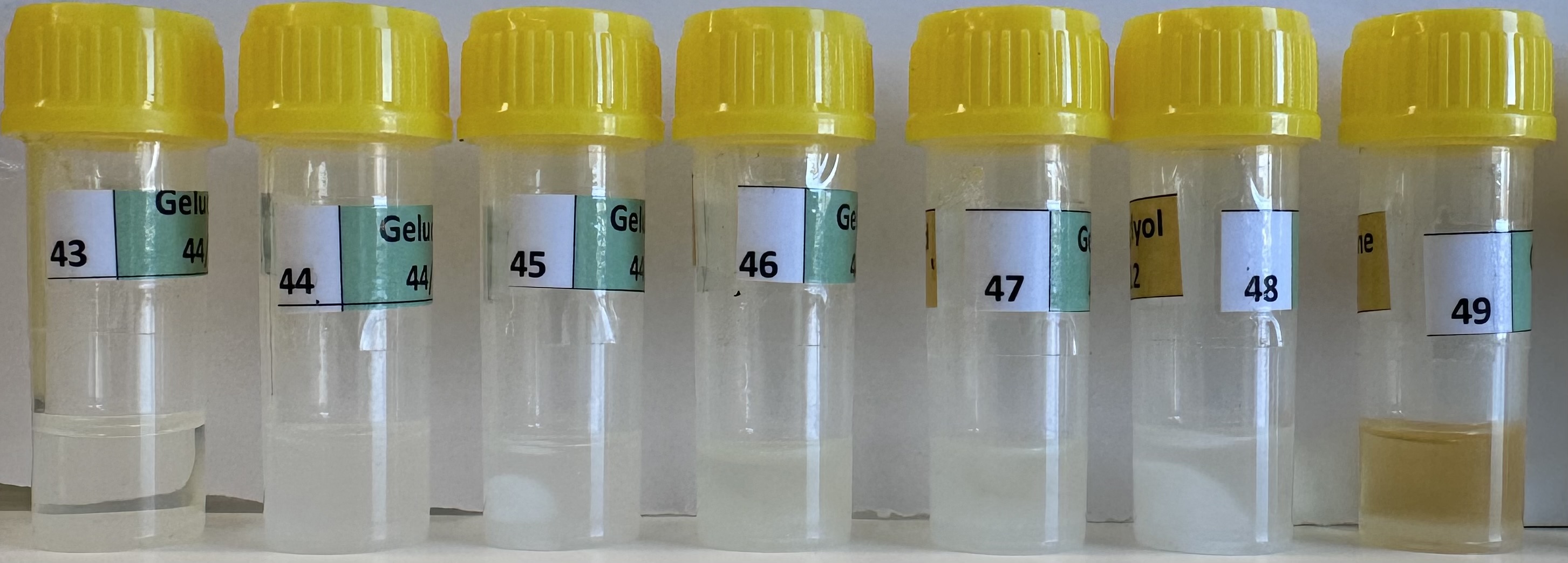


**Day 14**

**Day 0**


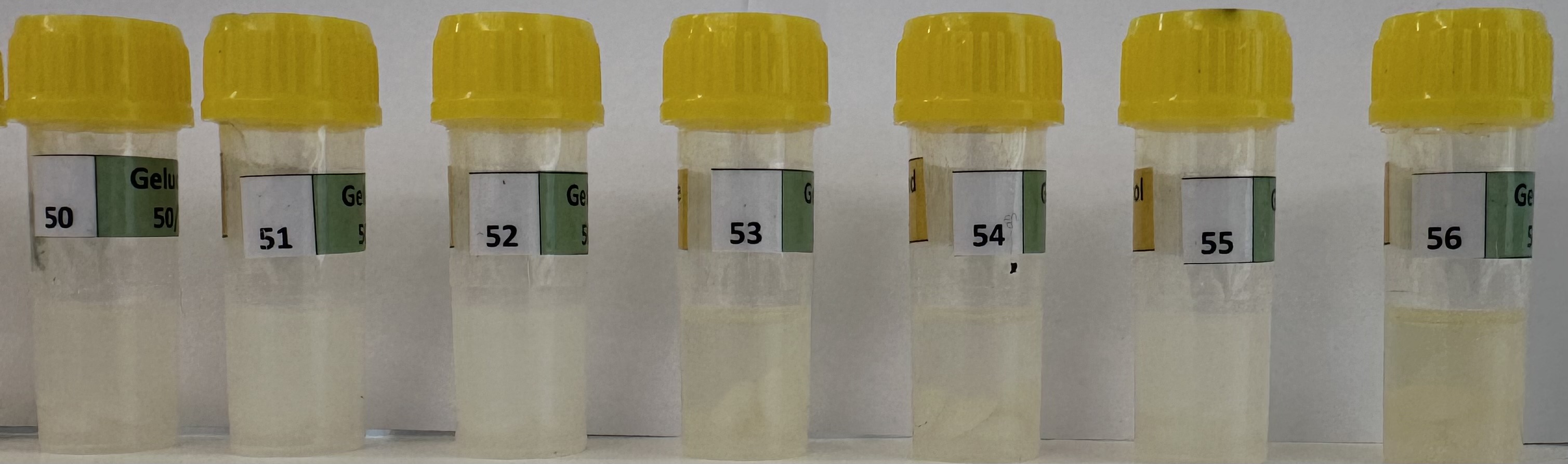


**Day 14**

**Day 0**


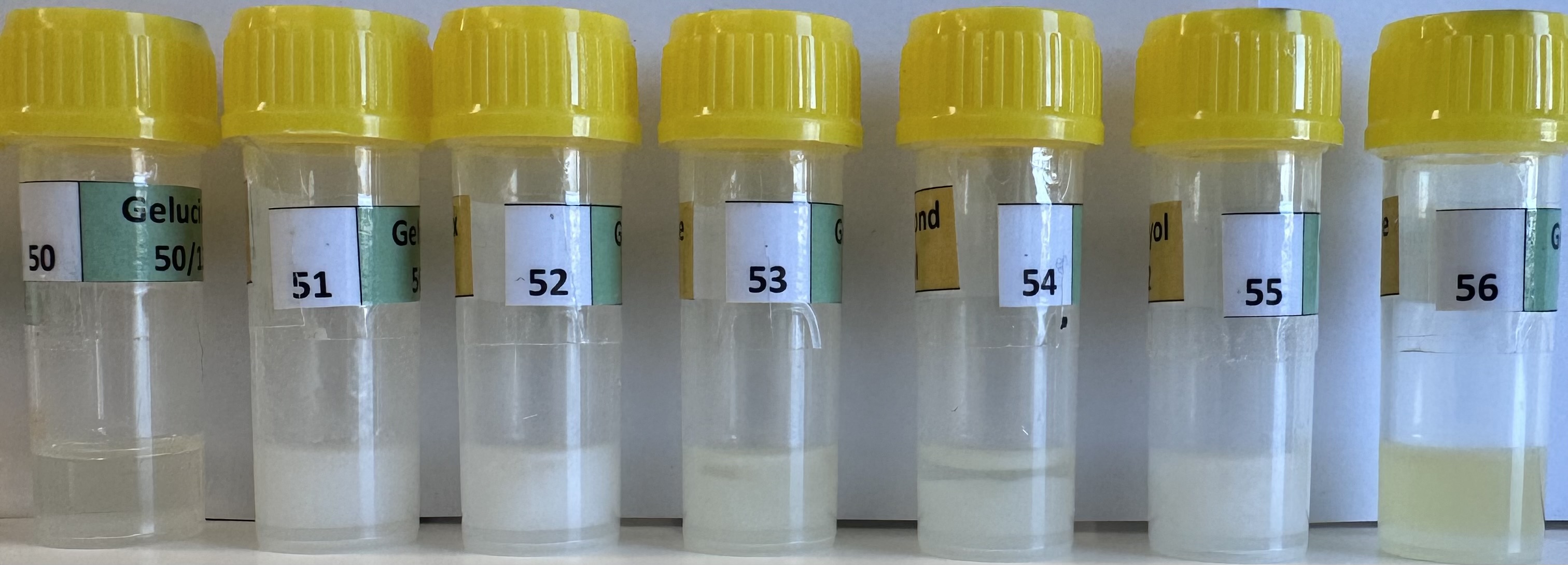


**Supplementary Fig. 1** Visual Stability Assessment of 56 Initial SNEDDS Formulations at Day 0 and After 2 Weeks of Accelerated Storage at 60°C During Pre-DoE Screening for CBD-PLC-SNEDDS Optimization


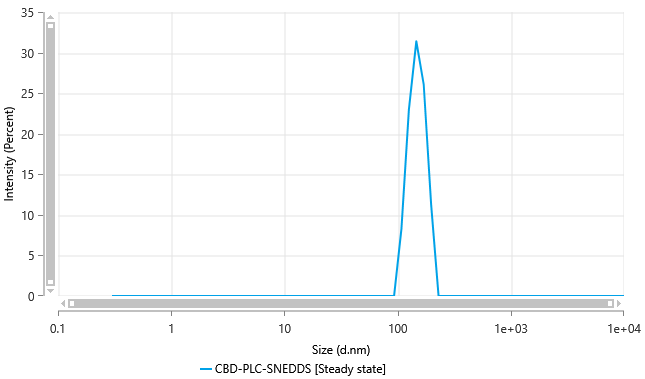


**Supplementary Fig. 2** Representative Particle Size Distribution of Optimized CBD-PLC-SNEDDS


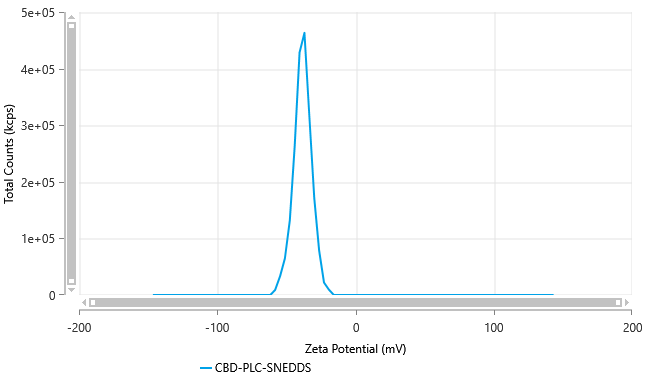


**Supplementary Fig. 3** Zeta Potential Distribution of Optimized CBD-PLC-SNEDDS


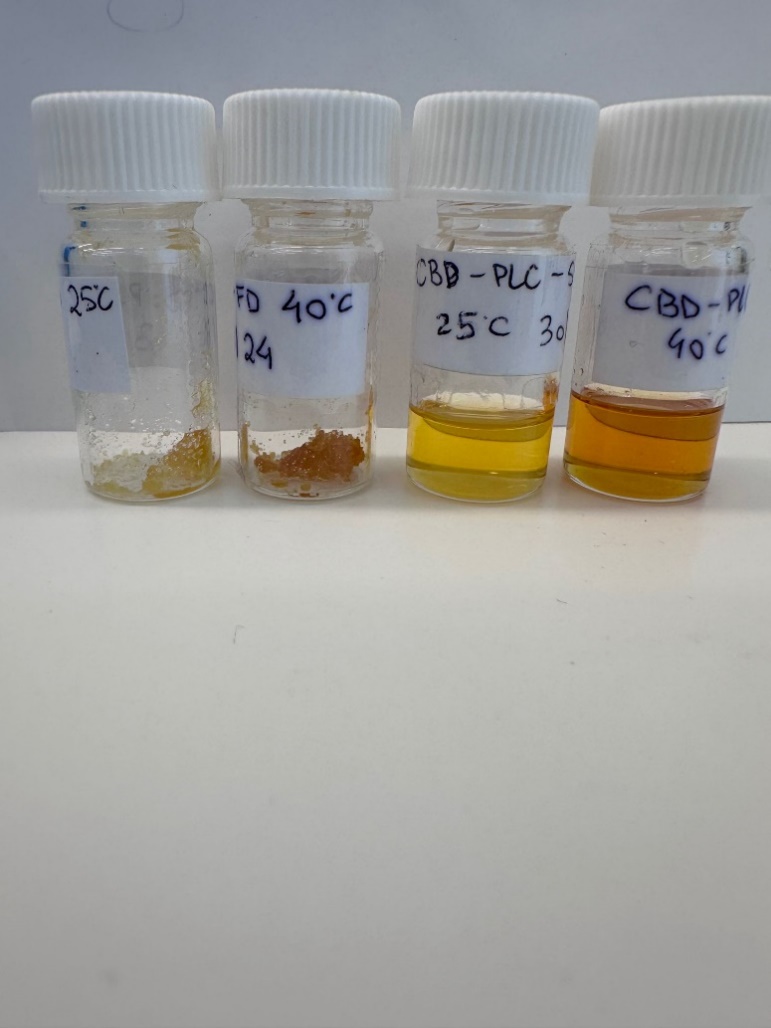


**Supplementary Fig. 4** Visual Appearance of CBD-PLC-SNEDDS Formulations after 4 Months of Storage at 25°C/60% RH and 40°C/75% RH (ICH Q1A(R2) Conditions)


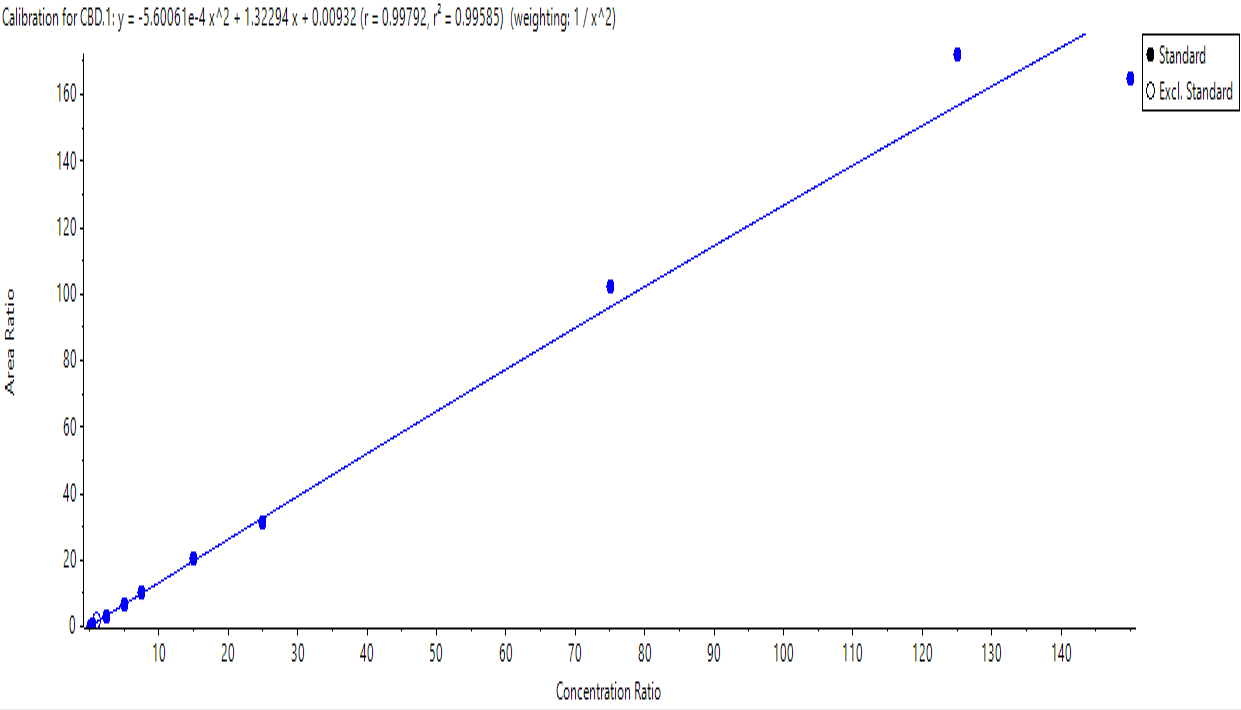


**Supplementary Fig. 5** Quadratic Calibration Curve for CBD in Rat Plasma Using LC-MS/MS (1–1500 ng/mL), Fitted with Model y=−5.601×104x2+1.323x+0.0093 y = -5.601 \times 10^4^ x^2^ + 1.323x + 0.0093 y=−5.601×104x2+1.323x+0.0093 (R² > 0.99)


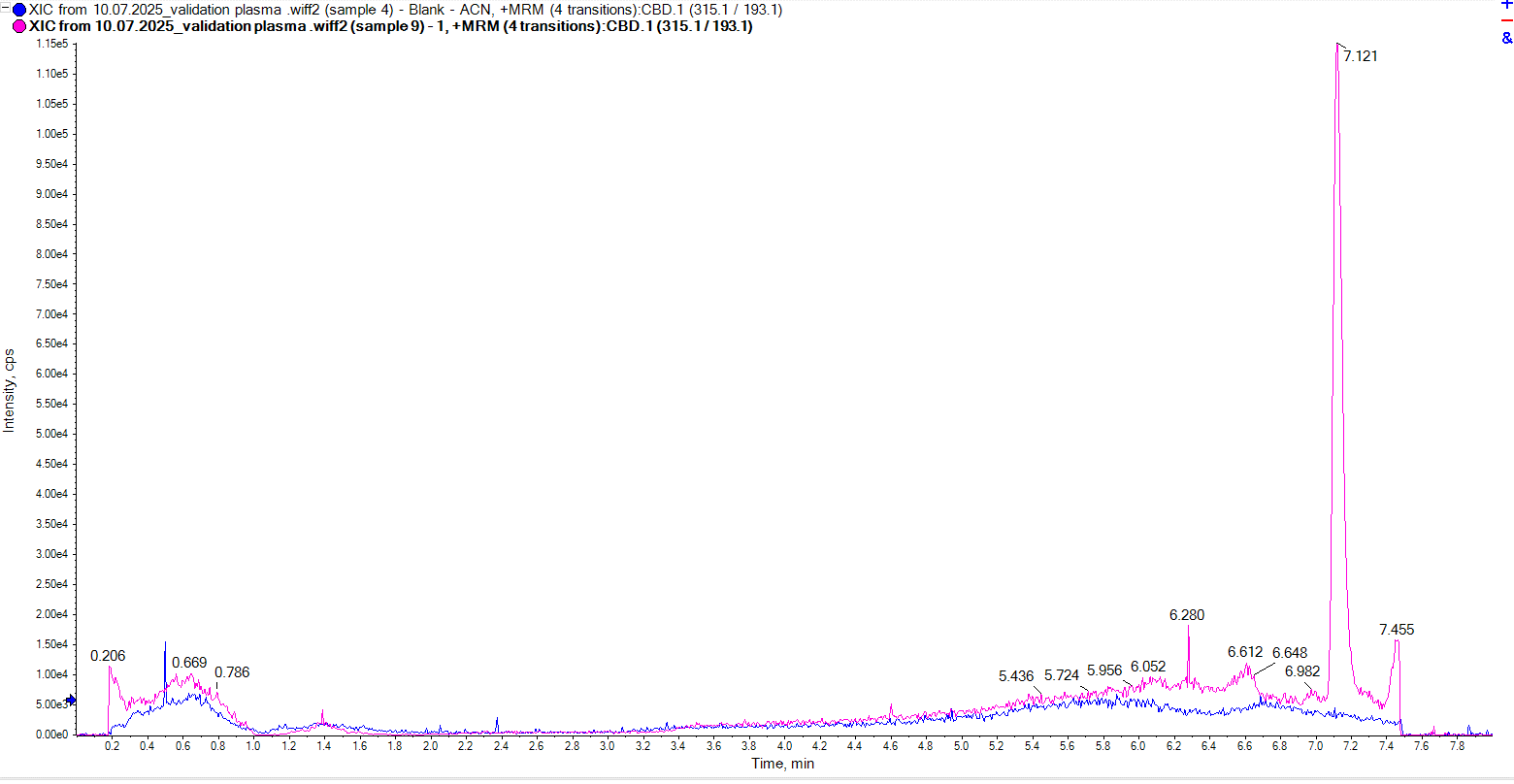


**Supplementary Fig. 6** Representative MRM Chromatogram of Cannabidiol (CBD) at Limit of Detection (LOD = 1 ng/mL) in Rat Plasma Extract, Validating LC-MS/MS Method Sensitivity


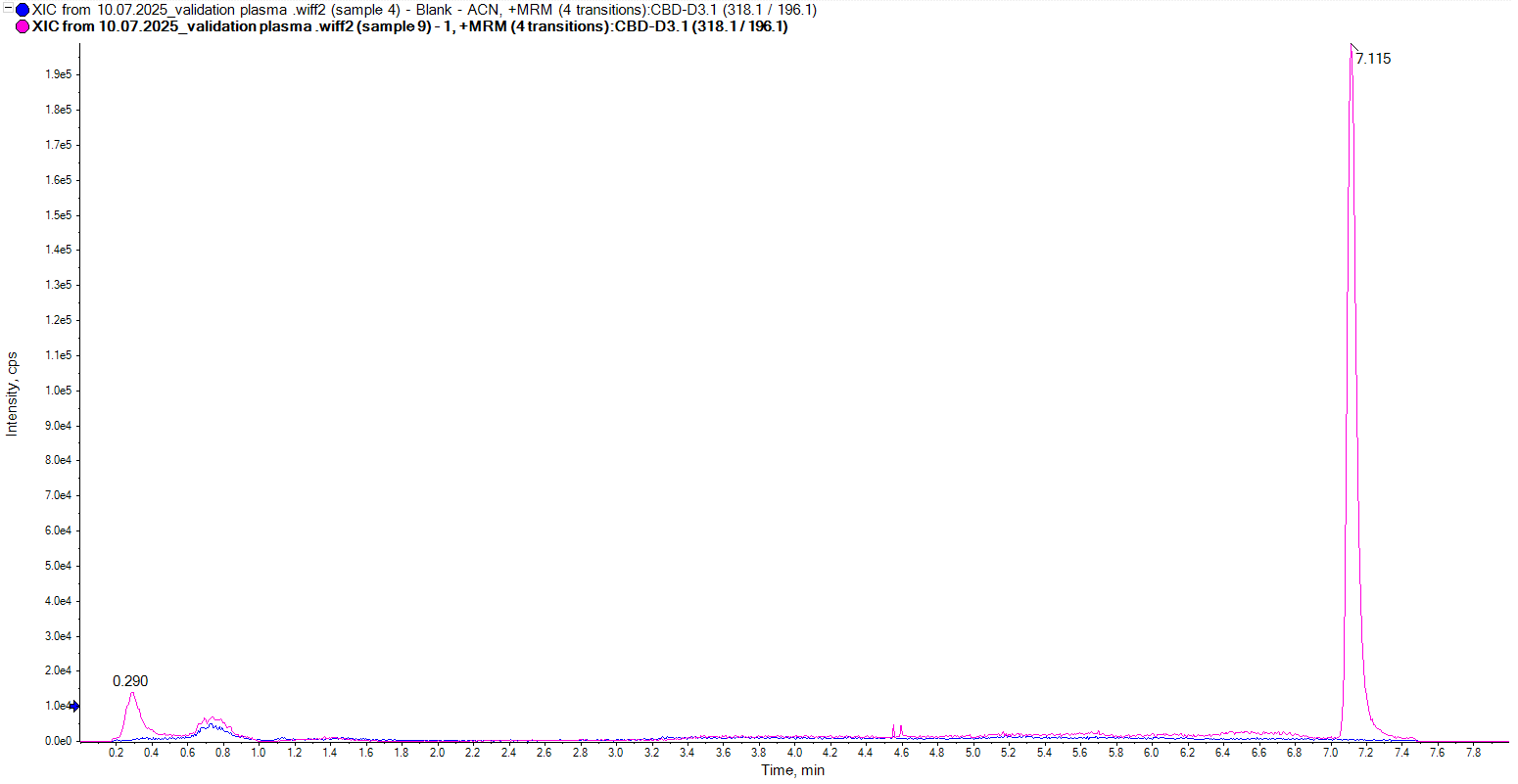


**Supplementary Fig. 7** Representative MRM Chromatogram of Internal Standard (CBD-D_3_) at LOD (1 ng/mL) in Blank Rat Plasma, Confirming Absence of Interference


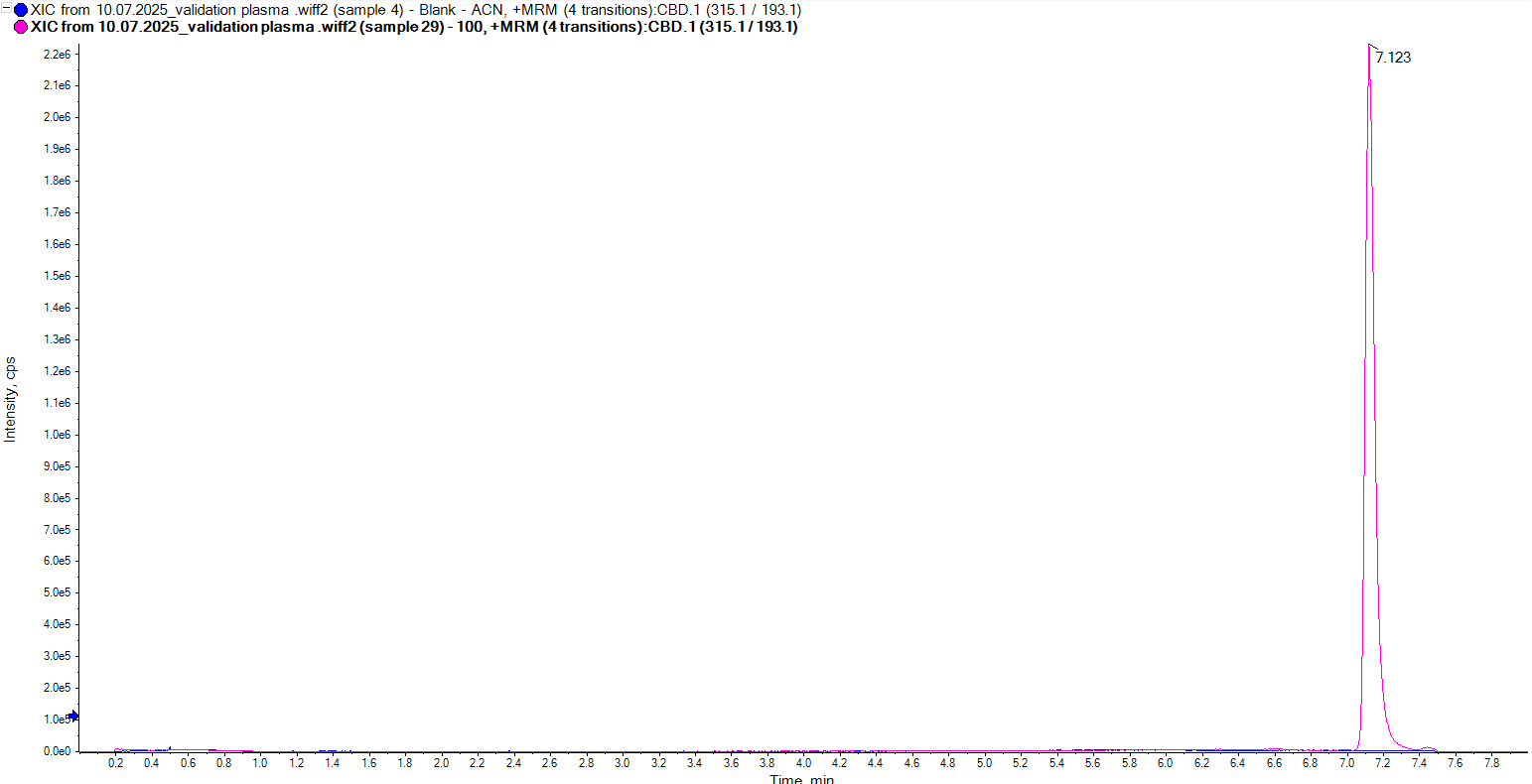


**Supplementary Fig. 8** Representative MRM Chromatogram of Cannabidiol (CBD) at 100 ng/mL in Rat Plasma, Demonstrating Method Selectivity and Peak Resolution


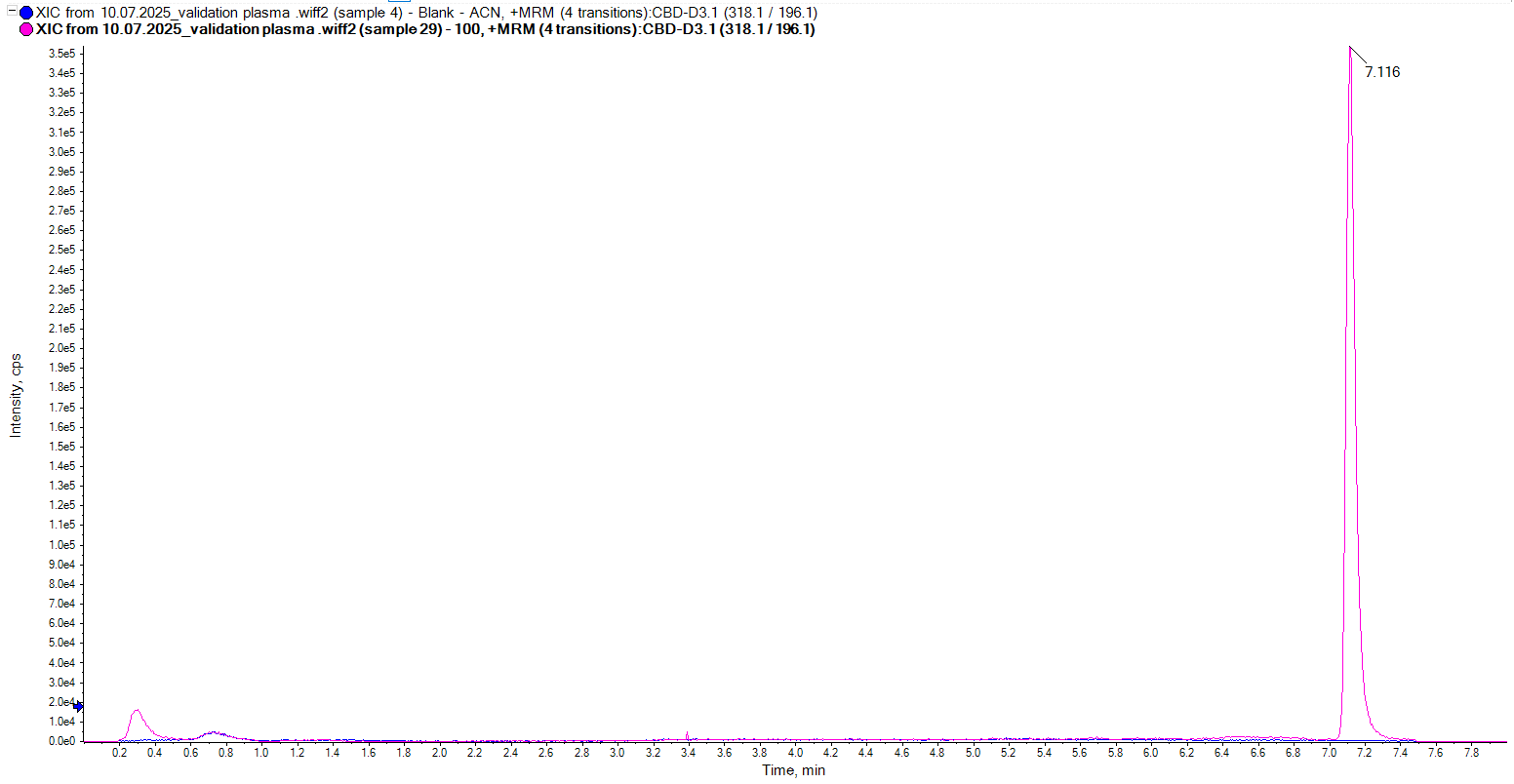


**Supplementary Fig. 9** Representative MRM Chromatogram of Internal Standard (CBD-D_3_) at 100 ng/mL in Blank Rat Plasma, Confirming Specificity and No Matrix Interference
